# Effects of AFQ056 on language learning in fragile X syndrome

**DOI:** 10.1172/JCI171723

**Published:** 2023-08-31

**Authors:** Elizabeth Berry-Kravis, Leonard Abbeduto, Randi Hagerman, Christopher S. Coffey, Merit Cudkowicz, Craig A. Erickson, Andrea McDuffie, David Hessl, Lauren Ethridge, Flora Tassone, Walter E. Kaufmann, Katherine Friedmann, Lauren Bullard, Anne Hoffmann, Jeremy Veenstra-VanderWeele, Kevin Staley, David Klements, Michael Moshinsky, Brittney Harkey, Jeff Long, Janel Fedler, Elizabeth Klingner, Dixie Ecklund, Michele Costigan, Trevis Huff, Brenda Pearson, NeuroNEXT FXLEARN Investigators

**Affiliations:** 1 Departments of Pediatrics, Neurological Sciences, and Anatomy & Cell Biology, Rush University Medical Center, Chicago, Illinois, USA.; 2 MIND Institute and Department of Psychiatry and Behavioral Sciences and; 3 MIND Institute and Department of Pediatrics, UCD, Sacramento, California, USA.; 4 Department of Biostatistics, University of Iowa, Iowa City, Iowa, USA.; 5 Department of Neurology, Massachusetts General Hospital, Boston, Massachusetts, USA.; 6 Division of Child and Adolescent Psychiatry, Cincinnati Children’s Hospital Medical Center, Cincinnati, Ohio, USA.; 7 Department of Psychology, University of Oklahoma, Norman, Oklahoma, and Department of Pediatrics, University of Oklahoma Health Sciences Center, Oklahoma City, Oklahoma, USA.; 8 MIND Institute and Department of Biochemistry and Molecular Medicine, UCD, Sacramento, California, USA.; 9 Department of Human Genetics, Emory University School of Medicine, Atlanta, Georgia, USA.; 10 College of Nursing and; 11 Departments of Pediatrics and Communication Disorders and Sciences, Rush University Medical Center, Chicago, Illinois, USA.; 12 Department of Psychiatry, Columbia University, and New York State Psychiatric Institute, New York, New York, USA.; 13 The NeuroNEXT FXLEARN Investigators group is detailed in Supplemental Acknowledgments.

**Keywords:** Clinical trials, Neuroscience, Neurodevelopment, Translation

## Abstract

**BACKGROUND:**

FXLEARN, the first-ever large multisite trial of effects of disease-targeted pharmacotherapy on learning, was designed to explore a paradigm for measuring effects of mechanism-targeted treatment in fragile X syndrome (FXS). In FXLEARN, the effects of metabotropic glutamate receptor type 5 (mGluR5) negative allosteric modulator (NAM) AFQ056 on language learning were evaluated in 3- to 6-year-old children with FXS, expected to have more learning plasticity than adults, for whom prior trials of mGluR5 NAMs have failed.

**METHODS:**

After a 4-month single-blind placebo lead-in, participants were randomized 1:1 to AFQ056 or placebo, with 2 months of dose optimization to the maximum tolerated dose, then 6 months of treatment during which a language-learning intervention was implemented for both groups. The primary outcome was a centrally scored videotaped communication measure, the Weighted Communication Scale (WCS). Secondary outcomes were objective performance-based and parent-reported cognitive and language measures.

**RESULTS:**

FXLEARN enrolled 110 participants, randomized 99, and had 91 who completed the placebo-controlled period. Although both groups made language progress and there were no safety issues, the change in WCS score during the placebo-controlled period was not significantly different between the AFQ056 and placebo-treated groups, nor were there any significant between-group differences in change in any secondary measures.

**CONCLUSION:**

Despite the large body of evidence supporting use of mGluR5 NAMs in animal models of FXS, this study suggests that this mechanism of action does not translate into benefit for the human FXS population and that better strategies are needed to determine which mechanisms will translate from preclinical models to humans in genetic neurodevelopmental disorders.

**TRIAL REGISTRATION:**

ClincalTrials.gov NCT02920892.

**FUNDING SOURCES:**

NeuroNEXT network NIH grants U01NS096767, U24NS107200, U24NS107209, U01NS077323, U24NS107183, U24NS107168, U24NS107128, U24NS107199, U24NS107198, U24NS107166, U10NS077368, U01NS077366, U24NS107205, U01NS077179, and U01NS077352; NIH grant P50HD103526; and Novartis IIT grant AFQ056X2201T for provision of AFQ056.

## Abstract 2

Despite extensive evidence of pre-clinical efficacy, the FXLEARN trial did not find benefit of AFQ056 for language learning in young children with fragile X syndrome.

## Introduction

Fragile X syndrome (FXS) is the most common known single-gene cause of intellectual disability (ID) and autism spectrum disorder (ASD), with an estimated prevalence of about 1:4,000 to 5,000 (1). FXS is an X-linked disorder caused by large expansions in fragile X messenger ribonucleoprotein-1 (*FMR1*, >200 CGG repeats), termed the “full mutation,” which results in methylation and transcriptional silencing of *FMR1* with consequent loss or substantial reduction in expression of the corresponding protein (FMRP) (2). FMRP is an mRNA-binding protein that regulates dendritic translation of many proteins critical for synaptic function and plasticity (3). Reduction or loss of FMRP results in dysregulated synaptic protein synthesis and impaired synaptic plasticity, learning, and cognition from birth (4).

Males with FXS typically have ID ranging from mild to severe (4). The average IQ in adult males is 40 to 50. In females, production of FMRP from the normal X chromosome in a percentage of cells results in a milder and variable phenotype marked by ID (25%), learning problems without ID, or normal cognition (5). In FXS, IQ and standard scores measuring adaptive behavior decline with age during childhood and adolescence due to failure to keep pace with typical development (6). Challenging behaviors (e.g., social avoidance, self-injury, hyperactivity, aggression) are common and affect family quality of life (7). About 50% of males and 20% of females with FXS meet ASD criteria (8). Although psychopharmacologic management of behavioral symptoms is often employed in FXS (9), these medications are rarely fully effective (10). There is no treatment for the underlying cognitive impairment in FXS, resulting in high family and societal costs for long-term care (7). Thus, targeted treatment of the underlying neurobiology for improving cognitive and developmental deficits associated with FXS represents an area of high unmet need (11, 12).

An extensive body of preclinical research has supported metabotropic glutamate receptor type 5 (mGluR5) negative allosteric modulators (NAMs) as potential disease-modifying agents (3, 4, 11) that target a neural mechanism of excess mGluR5 signaling in the absence of FMRP (13). The *Fmr1* KO mouse and cellular models have been used to confirm that FMRP regulates dendritic protein translation in response to synaptic activation by group 1 metabotropic glutamate receptors (mGluR1 and mGluR5) (3). In the normal state, activation of these receptors results in signaling through ERK- and mTOR-dependent signaling pathways, ultimately resulting in loss of FMRP repressor function at the ribosome and a subsequent pulse of new protein synthesis (14, 15). Precise control of translation and local levels of multiple key synaptic proteins regulated by FMRP is critical for maintenance of normal synaptic plasticity, including long-term potentiation (LTP) and long-term depression (LTD) (14, 16), dendritic spine morphology, and resulting cognition and behavior. In the absence of FMRP, there are abnormal levels of synaptic proteins usually controlled by FMRP, resulting in immature elongated dendritic spines (14, 16), abnormal spine density, abnormal synaptic plasticity, including enhanced hippocampal and cerebellar mGluR-activated LTD, impaired LTP in hippocampus, cortex, and amygdala, and abnormal epileptiform discharges (14, 16). The morphological and synaptic plasticity abnormalities found in the *Fmr1* KO mouse and the *Drosophila* model of FXS are associated with numerous cognitive, behavioral, and electrophysiological phenotypes (4, 14, 16).

The preclinical literature supporting the use of mGluR5 NAMs in FXS is the largest body of research on a treatment target in FXS or any neurodevelopmental disorder (NDD), including more than 50 papers from more than 20 laboratories reporting pharmacological reversal of over 30 molecular/cellular, synaptic, electrophysiological, behavioral, cognitive, and physical phenotypes in FXS mouse and *Drosophila* models using 1 of 4 mGluR5 NAMs (fenobam, CTEP, AFQ056, and MPEP) or genetic mGluR5 reduction (reviewed in refs. 4, 11, 14, 16; see Supplemental Table 1 for details on phenotype categories reversed and specific references; supplemental material available online with this article; https://doi.org/10.1172/JCI171723DS1). In multiple studies, correction of plasticity/morphology in FXS animal models by mGluR5 NAMs was coupled with normalization in learning paradigms, with evidence of more dramatic and complete reversal in younger (preadolescent) animals when compared with adults (17, 18), and with longer treatment duration (18, 19). Behavioral phenotypes (e.g., anxiety, perseverative behavior) were reversed in many studies, but these were often not as robust and reproducible as synaptic phenotypes and were dependent on experimental paradigm variables (e.g., mouse strain, environment, laboratory). Although results in mouse models indicated that learning and cognition would be the optimal primary targets for mGluR5 NAMs in FXS, and learning is the core problem in FXS, the regulatory environment and lack of good cognitive measures for FXS precluded a focus on learning in the initial human trials.

Preclinical work led to study of mGluR5 NAMs in humans with FXS, initially through a phase 1b pharmacokinetic (PK)/pharmacodynamic PD single-dose study of fenobam showing normalization of prepulse inhibition deficits (20). Following this, several phase 2a safety studies were run with mGluR5 NAMs from Novartis (AFQ056, mavoglurant) and Roche (RO4917523, basimglurant). Based on phase 2a signals (21), 3 multinational phase 2b studies (AFQ056, age 18+, *n* = 162; AFQ056, ages 12–17, *n* = 140; RO4917523,age 14+, *n* = 183) (22, 23) were conducted with primary outcomes of the Aberrant Behavior Checklist–Community Edition (FXS scoring algorithm, ABC_FX_, ref. 24, Novartis) and the Anxiety, Depression and Mood Scale (ADAMS, ref. 23, Roche). All 3 large-scale studies found large placebo effects, and none showed efficacy for drug over placebo. A substudy (*n* = 57) conducted within the Novartis phase 2b trials appeared to confirm target engagement, showing improvement in the AFQ056-treated group relative to placebo in performance-based measures, including eye-gaze behaviors during an eye-tracking paradigm (25) and correct answers and reduction of omissions in the Go-No Go task of the Kiddie Test of Attentional Performance (KiTAP) (26).

These trials did not answer the critical question regarding the efficacy of mGluR5 NAMs in FXS. Questions about research methods remained, including (a) whether observable behavior was an adequate clinical end point or whether the core deficits of development/cognition needed to be measured; (b) whether trials in adolescents and adults were optimal compared with intervention early in the course of the disease when plasticity is greater; (c) whether 3 months treatment duration is long enough to see clinically meaningful changes commensurate with disease modification; (d) whether a learning intervention needed to be coupled with drug administration to see effects on plasticity and learning, especially in a short time frame; (e) whether the large placebo effects seen on behavioral measures in the Roche and Novartis trials masked treatment or potential subgroup effects; and (f) whether the fixed dosing schedule in these trials prevented participants from showing optimal responses given variable interperson sensitivity to the drugs.

**Figure 1:**
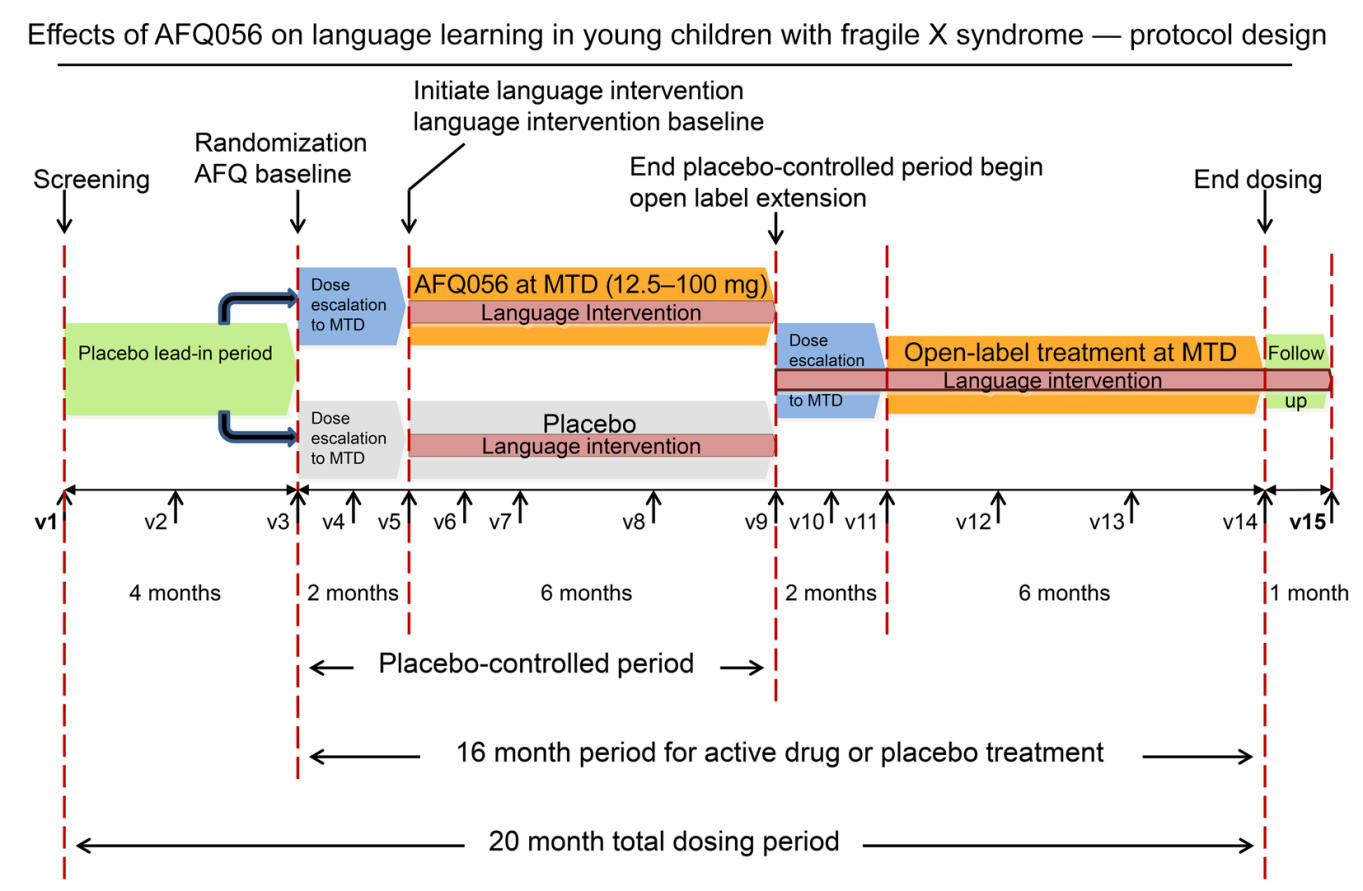
FXLEARN protocol design. v1, visit 1.

The present study, the FXLEARN trial (ClinicalTrials.gov NCT02920892; Figure 1), was designed to address the methodological concerns of prior studies and provide an answer to the question of efficacy of mGluR5 NAMs in FXS. This required an innovative trial design to study the effects on synaptic plasticity in the youngest children possible and was accomplished using a placebo-controlled, double-blind study of the potential impact of AFQ056 on language learning, a surrogate for neural plasticity, over an extended 8-month period. Drug/placebo administration was conducted in the context of a standardized parent-implemented language intervention (PILI) (27) provided to all enrolled subjects to accelerate learning. Additionally, the protocol utilized objective, performance-based outcome measures and biomarkers to limit placebo effects in evaluating treatment responses.

For FXLEARN, the mGluR5 NAM AFQ056 (Novartis) was chosen because a PK study had already been conducted in children with FXS, ages 3 to 11, allowing use of AFQ056 in children as young as 3 years. Additionally, a PET study showed AFQ056 binds to the target mGluR5 receptor and allowed estimation of receptor occupancy based on dose (28). Further, open-label extension (OLE) studies for participants from phase 2b placebo-controlled studies had evaluated long-term safety of AFQ056 in FXS for up to 3 years of treatment (29). Language learning was chosen as the focus for the study because it is highly relevant to children with FXS in the 3- to 6-year age range and a successful remotely delivered language learning intervention was available (27). Accordingly, the primary outcome measure, a centrally scored videotaped observational communication measure, the Weighted Communication Scale (WCS), assessed language learning to maximize the likelihood of observing of the specific change that would potentially be enhanced by the language intervention.

## Results

### Study enrollment.

**Figure 2:**
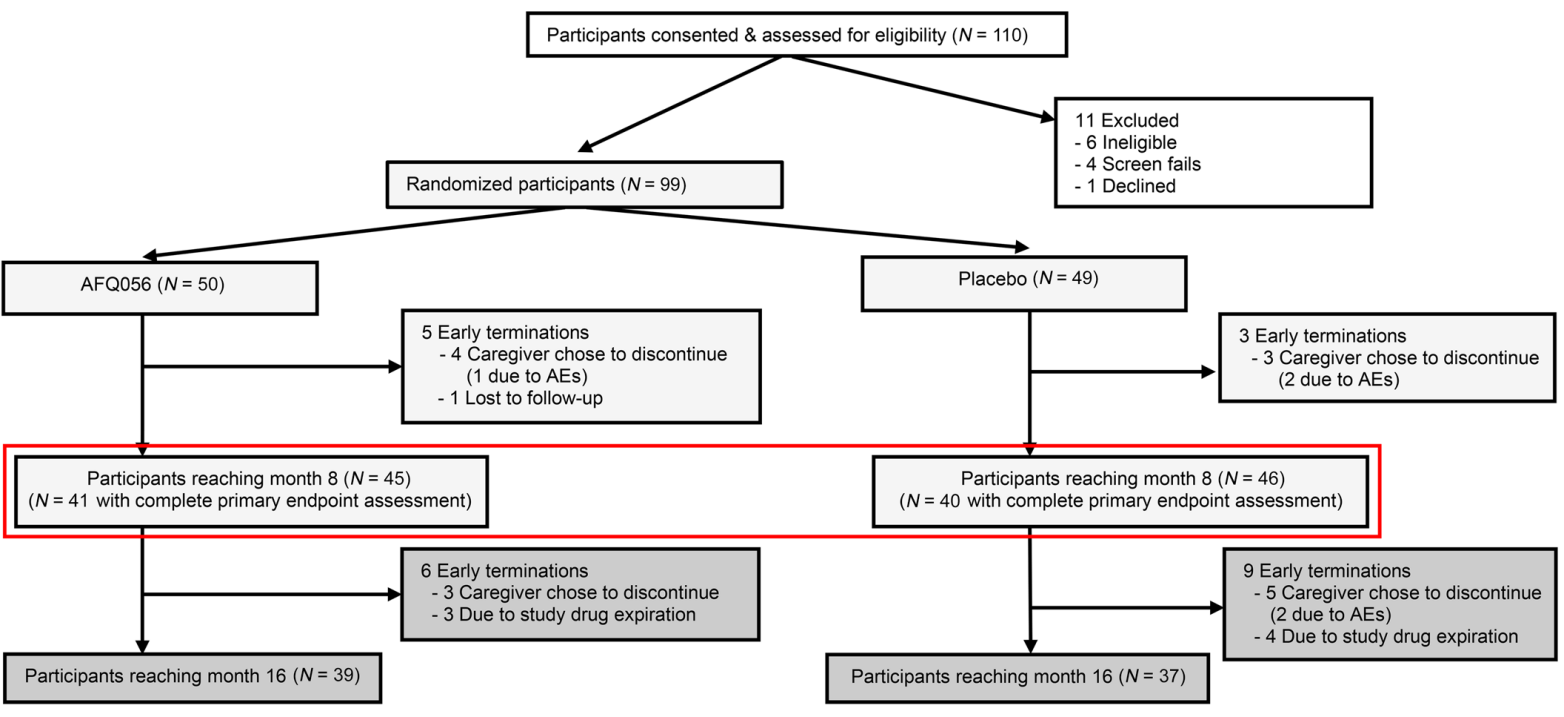
CONSORT diagram. FXLEARN consented 110 participants and randomized 99 to AFQ056 (*n* = 50) or placebo (*n* = 49). There were 8 early terminations during the randomized period and 15 during the open-label period. The red box shows the groups at the end of the randomized period, on which primary outcomes were based.

The FXLEARN trial enrolled 110 participants at 13 sites (Figure 2) between September 2017 and March 2020. Of these, 99 were randomized (6 ineligible, 4 screen failed, 1 withdrew) between January 2018 and July 2020. The last participant visit was in September 2021. Fifty participants were randomized to AFQ056 and 49 to placebo. Of these, 5 participants on AFQ056 discontinued (1 was due to behavioral side effects, 3 chose to discontinue in the absence of side effects [2 family burden, 1 needed prohibited medication], and 1 was lost to follow-up). Three participants on placebo discontinued (2 were due to behavioral side effects and 1 chose to discontinue in the absence of side effects). This left 45 participants on AFQ056 and 46 on placebo who completed the placebo-controlled period (Figure 2). Of these 91 participants, 89 enrolled in the OLE and 76 completed the OLE (7 withdrew and 6 stopped the OLE early due to drug expiration).

### Characteristics of the study groups.

Baseline demographics of the randomized child study participants and their parents delivering the language intervention, as well as clinical characteristics of the participants including baseline performance on study measures and use of nonstudy medications and standard-of-care therapies, did not differ between the 2 groups (Table 1 and Table 2). There were no significant differences between groups in the change in WCS scores, or on the Clinical Global Impression–Improvement (CGI-I) score for overall function during the placebo lead-in period.

### Study conduct and dosing comparisons between the study groups.

There was a nonsignificant trend toward better participation in PILI during the study for the parents of children in the AFQ056 group (Table 3). There were no significant differences in other parent fidelity measures for PILI between the placebo and AFQ056 groups (Table 3), suggesting a similar “dose” of the language intervention between groups. The distribution of the maximum tolerated dose (MTD) for drugs between groups was similar, with numerically but not significantly more placebo-treated participants reaching the maximum 100 mg twice a day (BID) dose (79%) than AFQ056-treated participants (69%) (Table 3). The distribution of protocol deviations, early terminations, and completion rate for all measures after randomization (88% across both groups) was not significantly different between groups. The placebo and AFQ056 groups were overall very similar in all parameters of study performance.

### Primary outcome.

**Figure 3:**
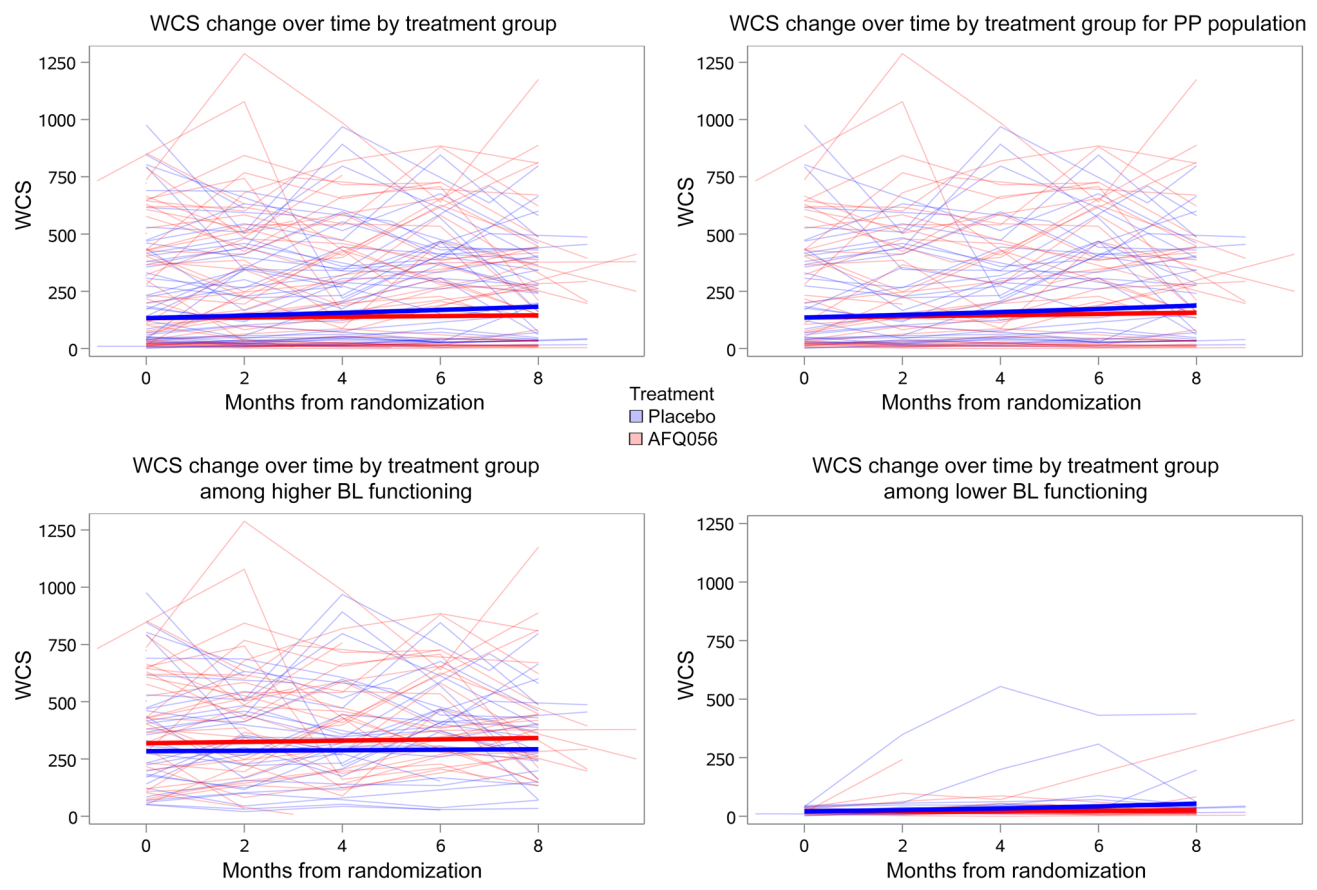
WCS score change over time. Shown for ITT (upper left), per protocol (PP) (upper right), ITT low functioning (<50 WCS, lower right) and high functioning (≥50 WCS) (lower left) groups. BL, baseline.

The primary outcome, change in WCS total score (log1_0_ scale) across the 8-month placebo-controlled period in the intent-to-treat (ITT) (*n* = 50 AFQ056, 49 placebo) analysis, was not significantly different between the AFQ056 and placebo groups (Figure 3 and Table 4). The placebo group showed a significant increase (improvement) in WCS score (0.14, 90% CI 0.05 to 0.22; *P* = 0.01) during the 8 months, whereas the AFQ056 group did not. A sensitivity analysis performed with the per-protocol population eliminated 12 and 14 participants in the AFQ056 and placebo groups, respectively, due to major protocol deviations or lack of PK samples to confirm presence or absence of drug. There was no significant difference between change in WCS total score across the 8-month placebo-controlled period in the per-protocol population (*n* = 38 AFQ, 35 placebo), and again, the placebo group but not the AFQ056 group showed a significant increase in WCS score (0.14, 90% CI 0.05 to 0.24; *P* = 0.01). Analyses based on 4 additional models, observed baseline only, last observation carried forward (LOCF), multiple imputation, and pattern mixture, all yielded results similar to those of the primary ITT and per-protocol analyses.

Further investigation of the unexpected result of lack of improvement in WCS total score in 8 months in the AFQ056-treated group revealed that when groups were split based on baseline WCS score into low versus high communication skills (<50 and ≥50, respectively), there was a statistically significant interaction (*P* = 0.02), suggesting that the effect of treatment differed by baseline functional status. Indeed, there was no difference in change in WCS between high communication skill placebo and AFQ056 groups, but a significant difference in WCS change in the lower communication skills group, such that the placebo group improved significantly more than the AFQ056 group (–0.35, 90% CI –0.56 to 0.13; *P* = 0.008) (Figure 3 and Table 4). Because it was thought that this effect might be mediated by behavioral side effects in the AFQ056 group leading to worse performance on the WCS, ABC_FX_ irritability and ABC_FX_ hyperactivity scores were also compared between AFQ056 and placebo groups. Although not statistically significant, there was a trend toward improvement in the placebo group for these scores, but not in the AFQ056 group during the 8-month placebo-controlled period (Table 4).

### Secondary outcomes.

Analyses of the secondary outcomes (Table 4) showed no significant difference in change during the 8-month placebo-controlled period in the Mullen Scales of Early Learning (MSEL) developmental quotient (DQ), MSEL Expressive Language raw score, Vineland Adaptive Behavior Scale–Version 3 (Vineland-3) adaptive behavior composite, Vineland-3 communication raw score, Preschool Language Scale–Version 5 (PLS-5) Expressive Communication raw score, or number of words on the MacArthur-Bates Communication Developmental Inventory (CDI) between AFQ056 and placebo groups. There was also no significant difference in the fraction of responders on the CGI-I for Overall Function score between groups. Both groups showed significant increases over the 8 months in the MSEL Expressive Language raw score (*P* < 0.01) and the PLS-5 Expressive Communication raw score (*P* < 0.01) and nonsignificant increases in the number of words on the MacArthur-Bates CDI. The AFQ056 group showed the expected significant decrease in DQ on the MSEL over the 8 months (*P* < 0.01), but the placebo group did not. When the cohorts were fractionated by functional level on the WCS, as above, the lower functioning group on AFQ056 did not show an increase in the MSEL Expressive Language raw score, while the lower functioning group on placebo and the higher functioning group on AFQ056 and on placebo all did show an increase in Expressive Language raw score. This supported the result from the WCS in which the low functioning group on AFQ056 did not show language progress.

### Safety outcomes.

During the placebo-controlled period, there was only 1 serious adverse event (SAE), in a placebo participant hospitalized for atypical pneumonia, deemed unanticipated, but not related to study treatment by the medical safety monitor. Ninety percent of participants in each AFQ056 and placebo group had an adverse event (AE) (Table 5). Most of these were typical childhood gastrointestinal, respiratory, or ear infections as well as insomnia or exacerbations of behavioral problems expected in FXS. No AEs showed a significantly different frequency between the AFQ056 and placebo groups.

### Safety in OLE.

In the OLE, all participants were treated with AFQ056 and the language-learning intervention for 8 months (Figure 1). The MTD reached across the entire group showed a similar distribution to that seen in the AFQ056 group during the placebo-controlled period, with 66% reaching the full 100 mg BID dose. There was 1 SAE, an episode of croup, felt to be unrelated to AFQ056. Rates and types of AEs in the OLE were similar to those in the placebo-controlled period (Supplemental Table 2), although in the OLE there were more AEs related to irritability and insomnia in the group that had been on placebo during the placebo-controlled period. Thus, these AEs may ameliorate over time on the drug or it is possible that dose reduction had resolved them before starting the OLE for those on AFQ056 in the placebo-controlled period. Additional analyses indicated that insomnia tended to resolve in the OLE, but irritability, if seen, tended to persist, making this symptom difficult to sort out from disease course in FXS.

## Discussion

The results of the FXLEARN trial demonstrate that treatment with AFQ056, an mGluR5 NAM, does not produce benefits for language learning and development in young children with FXS. FXLEARN was designed to address methodological concerns after the initial phase 2b trials of mGluR5 NAMs conducted by Roche and Novartis in adolescents and adults with FXS. Thus, the study focused on young children with greater neural plasticity, used objective measures less prone to placebo effect, optimized dosing, provided a longer treatment duration to give time for learning to occur, and added a standardized learning intervention to try to accelerate learning and amplify a potential drug effect. Learning was a focus of this study because the most robust and reproducible phenotype corrections by mGluR5 NAMs in FXS animal models were abnormalities/deficits in synaptic function and plasticity.

FXLEARN had several methodological strengths to increase confidence in the results. There was a high percentage of participant retention, no placebo effects on the WCS in the placebo lead-in, well-matched placebo and active groups for demographics and clinical characteristics, and rigorous data completeness, particularly for a study concluding during the COVID-19 pandemic. Despite the methodological rigor and design innovations, no differences were seen in change over time between the AFQ056 and placebo groups for the primary end point, the WCS score, or any of the key secondary end points. Nonetheless, significant improvement was observed on the WCS in the placebo group, but not the AFQ056 group, and significant improvement in language was also observed, as measured by the MSEL and PLS-5 expressive communication raw scores, in both groups. Overall, children with FXS made language progress during the 8-month placebo-controlled period of the study; however, the AFQ056 group did not make more progress and, if anything, made less than the placebo group.

This unexpected result is not likely due to placebo effects, such as those commonly seen in FXS trials on behavioral-rating measures completed by caregivers (30), given the fact that the tests measuring language progress in the placebo group were performance-based observational measures scored by blinded coders and thus would not likely display a placebo effect. The reduced progress in the AFQ056 group appears to be driven by the children with FXS in the lowest communication skills quartile at baseline, with the placebo group showing improvement in language progress on the WCS in contrast with the AFQ056 group, whereas this differential outcome was not seen in the children with higher communication skills. The reason for this is unclear; however, it was unrelated to better use of the language intervention in the placebo group, as parental fidelity was similar and participation in PILI was (nonsignificantly) better in parents of the AFQ056 group. The differential placebo-AFQ056 effect in the group with lower language function may have been driven by a few outliers in the placebo group (Figure 3) who showed progress, whereas most of the children in the lower functioning placebo group did not. It is possible that there were behavioral issues limiting either the ability of the children in the AFQ056 group to benefit from PILI or to perform optimally during the interactions used to compute the WCS. This hypothesis is supported by the fact that ABC_FX_ Hyperactivity and ABC_FX_ Irritability subscale scores improved numerically (albeit not significantly) in the placebo group but not in the AFQ056 group. There was, however, no increased rate of behavioral AEs in the AFQ056 group. It is possible that behavioral abnormalities not considered as AEs differentiated the groups and contributed to inhibition of efficacy of PILI in the AFQ056 group. Fidelity of PILI and behavioral impacts will be further addressed in future analyses.

There were no significant safety concerns associated with AFQ056 and no significant group differences in reported AEs even for irritability, insomnia, or behavioral activation during the placebo-controlled period. The only differential signal in AEs between drug and placebo groups was the emergence of irritability and insomnia AEs in the participants who switched from placebo to drug in the OLE. Review of the time course of these AEs suggested that insomnia tended to resolve with more time on the drug, potentially due to dose reduction or tolerance. Tolerance has been seen in the FXS mouse model, based on adaptations in neural signaling downstream of mGluR5 activation (31). Irritability did not resolve with increased time on drug, illustrating the difficulty in sorting out relationships to investigational drugs for behaviors often problematic in FXS. This could relate to differences in mGluR5-activated signaling in different parts of the brain, resulting in differential symptom responses (31–33). Drug reductions (Table 2) due to behavior while titrating to MTD in the placebo group emphasize the challenges in interpreting behavioral AEs in FXS.

FXLEARN was rigorous and well powered and not subject to any identifiable between-group biases. Therefore, we believe that the present study clearly answers the questions raised by problematic placebo effects and concerns about the ages of participants and limitations of (behavioral) outcome measures in the initial adolescent and adult mGluR5 NAM trials. The FXLEARN result, in combination with prior negative trials, indicates that reduction of mGluR5 activity does not improve cognitive or behavioral deficits in humans with FXS. Consequently, it raises concerns about translatability of animal-model findings in FXS. In 15 of the studies showing phenotype reversal with mGluR5 NAMs in FXS mouse models, including the studies using AFQ056, investigators were blinded to treatment groups and/or assessment of outcomes (Supplemental Table 1) and used littermates as controls chosen without obvious bias; 2 studies even used a true randomization scheme. Given the generally good quality of this preclinical work and the large volume of publications supporting the benefit of mGluR5 NAMs in mouse, fly, and rat models of FXS, the trial results reported here suggest that mGluR5-mediated responses in humans may have diverged evolutionarily from animal models and are not as important in mediating the neural effects of absence or marked reduction in FMRP or that the mGluR5 signaling system is differentially active in key brain regions in humans relative to rodents and thus less relevant to FXS. Differential functioning of mGluR5 signaling in different areas of the brain has been observed in the FXS mouse and rat models, leading to the suggestion that mGluR5 signaling is actually underactive in some areas of rat brain (32, 33). PET studies in adult males with FXS have shown reduced cerebral mGluR5 expression (34). If these areas of mGluR5 underactivity were more extensive in human FXS brain, then learning might actually be impaired by an mGluR5 NAM, and this could provide an explanation for the observation of less language learning in the AFQ056 group during the placebo-controlled phase of FXLEARN. Patterns of *Fmr1* expression during development have been shown to differ between rodent and primate models, including differing regulation of cortical nitric oxide synthase expression (35).

Certain neural-signaling pathways in FXS animal models may be better conserved and translate better to humans, while some have evolved to be less important and less translatable. In fact, recent studies have shown differential response of human iPS cell–derived neurons to mGluR5 NAMs compared with mouse neurons (36) and a lack of benefit of mGluR5 NAMs in human FXS iPS cell–derived cerebral organoid (37). Such human neural models may be helpful in predicting translatability of new disease-directed agents in FXS. Even with such studies, it is likely to be very difficult to predict which pathways and treatment targets will translate well to individuals affected by the disorder, so early phase target engagement studies with objective measures in FXS participants are particularly important before moving to large trials that may use participant, time, and monetary resources to no positive end (11, 38, 39). Also, it will be imperative to measure animal model phenotypes that can be directly translated into humans, such as electrophysiological measures (EEG) and other biomarkers applicable to both mouse and human studies in FXS (40).

This paper reports the primary and key secondary outcomes of FXLEARN. Separate analyses of the effects of PILI, including effects of the amount and fidelity of the PILI intervention on improvement in language, will be the subject of future analyses. Analyses of the impact of AFQ056 on blood biomarkers, eye tracking, resting EEG, and event-realted potentials (ERPs) and the impact of PK (drug levels) on trial outcomes, currently ongoing, may be quite informative (41). As is the case for many drugs acting on the central nervous system, there may be subpopulations of individuals with FXS that are drug responders, as predicted by *FMR1* mutation type, FMRP levels, electrophysiological parameters, or other biomarker responses, given that these parameters can predict clinical phenotype (42). There are precedents for subgroup responses in other trials (43) based on mutation type, and further analyses to evaluate these types of responses will be forthcoming.

FXLEARN will contribute substantially to the understanding of rates of development in FXS, as assessed by the measures used in the trial, as well as to the understanding of how these measures perform in children with FXS in the 3- to 7-year age range. These data will be valuable for informing future long-term intervention studies, such as gene therapy, and for powering future longer term trials. The remote WCS developed during the COVID pandemic will potentially provide a remote measure for future trials in young children with FXS or other NDDs to reduce travel burden. FXLEARN makes available many resources for the field, including manuals for ERP and eye-tracking standardization across sites, case report forms designed for history capture and AE monitoring in FXS, an anchored CGI-Severity/Improvement (CGI-S/I) score for young children with FXS, and procedures for remote PILI delivery.

Limitations of FXLEARN included some missing data, partly due to concluding the study during the COVID-19 pandemic. However, adjustments made for this in the analyses did not affect the study findings, so it is unlikely the findings were affected by missing data. The intensity of the study and collection of multiple measures in the clinic visits may have resulted in suboptimal participant performance, even though efforts were made to implement processes to avoid this, such as breaks and testing on multiple days. Some visit windows had to be widened due to the pandemic, which could have potentially affected data, although as well as can be evaluated, this does not seem to be the case.

In conclusion, this type of trial combining targeted medication treatment and language intervention addressed shortcomings of prior trials, but did not demonstrate evidence for a benefit of the mGluR5 NAM, AFQ056, on language learning in young children with FXS. These results are consistent with prior trials in adolescents and adults showing a lack of benefit for this mechanism of treatment in FXS (24, 26, 27). The complex and labor-intensive design of the present trial proved feasible to carry out with high methodological standards, despite the challenging population and interference by a pandemic. This was, to our knowledge, the first-ever large multisite trial studying the effects of a targeted drug treatment on learning in FXS and provides a model for implementing future studies to determine whether employing a learning intervention can amplify benefits of a drug targeting the underlying mechanism in FXS and other NDDs.

## Methods

### Study design.

FXLEARN used a double-blind, placebo-controlled, parallel-group, flexible-dose, forced-titration design with an embedded language-learning intervention (PILI) (Figure 1). A 4-month placebo lead-in period was followed by an 8-month placebo-controlled period (2-month dose optimization, 6-month stable dosing), in which participants were randomized 1:1 to AFQ056 or placebo. Participants started PILI (27) at the end of 2 months in the placebo-controlled period, after their doses were stabilized at the MTD, and continued with PILI throughout the remainder of the study. If caregivers chose to continue after the end of the placebo-controlled period, participants entered an 8-month (open label, 2-month dose optimization, 6-month stable dosing) extension period in which all participants were treated with active drug. After the OLE, there was a 1-month follow-up period. FXLEARN was conducted at 12 sites within the Network for Excellence in Neuroscience Clinical Trials (NeuroNEXT) network and at Rush University Medical Center, the principal investigator’s site (not a NeuroNEXT site).

### Participants.

Eligible participants were children aged 32 months to 6 years inclusive with FXS and an *FMR1* full mutation, who had a DQ of less than 75 calculated from the MSEL at screening, spoke English as the primary language at home, displayed some evidence of intentional communication, were on stable behavioral and other therapies for 30 days prior to starting the trial, and were on stable doses of chronic medications for 60 days prior to trial initiation. Participants were excluded if they were taking γ-aminobutyric acid (GABA) or glutamatergic medications, had a seizure within 6 months prior to screening, were using strong or moderate inhibitors or inducers of CYP1A1/2, CYP2C9/19, or CYP3A4, or had a concomitant medical illness or abnormal laboratory tests that would contraindicate study participation based on the investigator’s judgement.

### Study conduct.

Participants meeting entry criteria were started on 2.5 cc BID of placebo suspension after the screening visit and began the 4-month placebo lead-in period. The placebo lead-in served to control for placebo effects, but also to create a placebo/treatment-as-usual period prior to initiation of PILI to compare with the period of PILI use in the group later randomized to placebo as a way of assessing the effects of PILI without drug. Although participants as young as 32 months were enrolled in the study, because of time spent in the placebo lead-in, all subjects were 3 years of age or older before exposure to active drug.

At the end of the placebo lead-in, participants had baseline assessments and then were randomized 1:1 to AFQ056 or placebo. Randomization was stratified by age (3–4 years and 5–6 years). After randomization, there was a 2-month dose-titration period to find the MTD for each participant. This flexible dose design mimicked practice and considered interchild variability in drug levels and responsiveness. Dose titration to MTD (allowed doses were 12.5 to 100 mg [1.25–10 cc] BID) started at 25 mg (2.5 cc) BID and used a forced-titration (mandatory titration unless there were side effects) protocol with weekly titration and options for holding doses or dose reduction if side effects, such as insomnia, hyperactivity, or other CNS activation, occurred. The starting dose could be decreased to 12.5 mg BID if side effects occurred, but participants who could not tolerate 12.5 mg BID were withdrawn from the study. After the 2-month titration to MTD, participants had baseline assessments repeated prior to initiating PILI, after which they remained on a stable AFQ056/placebo dose for the next 6 months. At the end of the placebo-controlled period, all assessments were performed again, and participants had the option to enter the OLE. None of the doses of standard-of-care medications for behavior or nondrug therapy interventions in place at the screening visit were changed until the end of the placebo-controlled period.

Participants entering the OLE had their dose adjusted to 25 mg (2.5 cc) of AFQ056 twice a day (to start drug if in placebo group and a dose decrease for most participants in the AFQ056-treated group). Experience from a prior PK study suggested that this dose reduction would not likely produce withdrawal symptoms. Participants then underwent dose titration to MTD as in the placebo-controlled period, over up to 2 months, followed by 6 months of stable treatment. Participants continued PILI during the OLE phase. Participants again had all baseline assessments repeated at the end of the OLE and then tapered off AFQ056 if they were on a dose higher than 25 mg BID. If on 25 mg BID or less, they just discontinued AFQ056. Participants returned for follow-up assessments a month after the end of the OLE.

### AFQ056 study drug and placebo.

Study drug consisting of AFQ056 or matching placebo with identical flavoring was shipped as powder in bottles (500 mg AFQ056 per bottle) from Novartis to the University of Rochester NeuroNEXT Central Pharmacy where bottles were packaged into kits with 10 bottles each and shipped to research pharmacies at the sites. Bottles were labeled at the site research pharmacies. Placebo powder (during placebo lead-in), AFQ056 (500 mg) or placebo powder (during double-blind period), and AFQ056 (500 mg) powder (during OLE) in bottles were dispensed to parents/caregivers. The powder was dissolved in 50 cc of bottled water measured with a syringe to give a 10 mg/mL suspension. The powder-dissolving process for the first bottle to be used was demonstrated with the parent/caregiver in clinic, and subsequent bottles of AFQ056/placebo powder were dissolved by the parent or caregiver at home every 10 days or earlier, if needed, based on dose (stability of the suspension was estimated at 10 days). Documentation of the reconstitution at study visits and at home was maintained. Drug accountability was performed at all study visits.

In the placebo-controlled period, randomization occurred through an interactive web-response system that resulted in kit numbers containing placebo or AFQ056 being assigned to the participant. Participants and study staff were blinded to treatment assignments. Only the research pharmacy at each site was unblinded to treatment assignments to provide the correct kit numbers. Kits containing the projected number of bottles needed before the next visit were provided to the family. The study remained blinded until all participants completed all study procedures, and the clinical database was locked.

### PILI.

PILI was administered in the family home via video teleconferencing to the designated parent/caregiver for each participant by a speech-language pathologist (SLP) trained to fidelity on the intervention with videotapes and practice activities. After several distance-technology training sessions conducted in the weeks before PILI was to start, a didactic education session was administered at the start of every 4-week interval to the parent to provide a rationale and examples of the language-facilitating strategy to be practiced over the coming 4 weeks. This education session was followed by weekly clinician coaching, homework, and feedback sessions (described below). PILI was delivered to the parent in the home through a MacBook laptop computer and a Bluetooth “bug in the ear” headset (provided to the parent/caregiver by the study) equipped with the standardized distance video-teleconferencing software Skype for Business for Macs, Microsoft, version 16.29.42. Coaching, homework, and feedback sessions occurred weekly for the first 4 months, with each month being led off with a parent-education session introducing a new strategy and then monthly for the remainder of the study through the placebo-controlled portion and the subsequent OLE. The parent/caregiver was required to complete all 4 parent-education sessions; 41 of 48 (85%) combined coaching, homework, and feedback sessions during the first 4 months of PILI, and 25 of 33 (76%) combined coaching, homework, and feedback sessions during the subsequent 11 months.

PILI didactic sessions involved slide presentations with video examples. The coaching sessions involved real-time instruction and feedback to the parent through the Bluetooth-connected devices while the parent interacted with the child in a play-based format. The parent independently recorded and submitted homework practice sessions, which involved the parent implementing the language facilitation strategies being targeted that week in an interaction with the child. Parents uploaded the videorecorded homework to their clinical teams via an online file-sharing system. The SLP then provided feedback via videoconferencing, focusing on implementation of targeted strategies and management of child-challenging behaviors.

Throughout the intervention, parents were encouraged to use the strategies with their children in naturally occurring opportunities throughout the day. As a way of providing an estimate of parent mastery and use of the targeted strategies, parents reported how often they used the targeted strategies between contacts with the SLP, and they were graded by their SLP on their ability to deliver, and comfort with, the strategies during coaching, homework, and feedback sessions. Variability in parental rate of mastery and frequency of use of the targeted strategies was expected. By examining clinician-rated parental fidelity of implementation and parent-reported frequency of use, the effective dose of PILI received by the children enrolled could be examined in relation to child outcomes, allowing study of the language intervention separately from, and in combination with, AFQ056.

PILI was designed to maximize the extent to which parents engage in the types of verbally responsive interactions that have been well documented as facilitating language learning and use in children with typical and atypical development (44, 45).These interactions are characterized by frequent parent talk about the child’s focus of attention, contingent parental responses to child actions and communication, parental language slightly in advance of child language levels, affectively positive parental talk, and parent support for, and prompting of, child communication. Engaging in such behaviors is often difficult for parents of children with FXS because of the children’s developmental delays and cooccurring challenging behaviors (46). Thus, PILI attempts to teach parents specific strategies for engaging the child and creating a sustained, verbally responsive interaction. The specific strategies taught to parents were following the child’s focus of attention when communicating, responding contingently to child acts of communication, setting up conditions that prompt child communication, and encouraging the use of more advanced forms of language and communication. Several variations of this PILI have been shown to be effective in improving expressive language and communication in individuals with FXS who have varying ages and ability levels (27, 47–49).

### Safety assessments.

Information on AEs, vital signs, height, weight, physical and neurological exams, behavioral/psychiatric assessment, suicidality assessment, and concomitant medications was collected at every visit (Supplemental Table 3 shows full schedule of activities indicating when all assessments were collected). Safety was assessed by comparing the incidence, frequency, and severity of treatment-related AEs and SAEs between the treatment groups. Funduscopic exams, EKG, blood tests for hematology/chemistry, and urine dipsticks were monitored during the study.

### Autism status.

The Autism Diagnostic Observation Schedule–Second Edition (ADOS-2) (50) was administered prior to randomization to classify children with respect to ASD diagnosis.

### Efficacy assessments.

Efficacy assessments, including the WCS (primary outcome); MSEL, Vineland-3, PLS-5, MacArthur-Bates CDI, CGI-I (secondary outcomes); ABC_FX_, Visual Analog Scales (VASs), CGI-S, and biomarker assessments (exploratory outcomes), were administered to all participants at multiple times throughout the study (Supplemental Table 3).

The WCS (51–53) total score was the primary outcome measure for FXLEARN and was derived from a 22-minute semistructured examiner/child-play session. The play session was administered at screening, baseline, 2-, 4-, 8-, 10-, 12-, and 16 months, and follow-up visits by an SLP or psychologist trained to fidelity on administration of the measure. The same rater administered the measure to each child throughout the trial whenever possible. The sessions involved 12 minutes of structured play prompts designed to elicit a range of communicative behaviors (e.g., requesting, sharing of affect) and 10 minutes of free play with a standard set of toys. The play session utilized 3 sets of developmentally appropriate toys, and the toy sets were counterbalanced across participants and visits. The WCS was coded from videotapes of the sessions according to standard coding methods by coders trained to fidelity. The WCS reflects both the frequency of child-initiated intentional communication and the developmental level of the means by which the intention is communicated. In particular, coding of child-intentional communication was based on the occurrence of 3 classes of behavior: (a) gestures or nonword vocalizations during which the child coordinated attention between the message recipient and an object or salient event; (b) conventional gestures (e.g., distal points, head nods, pantomime) with attention to an adult; and (c) symbols (i.e., spoken words or signs) that were used in a nonimitative manner. The score was obtained by multiplying each intentional communication act by the following weights: nonverbal = 1; single symbol = 2; and multiple symbols = 3. Previous research has indicated that the weighted variable is more sensitive to change over time than the unweighted variable and that growth in the weighted variable (but not the unweighted variable) is linear, related to later levels of social impairment in younger siblings of children with ASD, and detects change in response to treatment (51–53).

If the participant used an augmentative device as the primary form of communication prescribed by a speech therapist, he/she was permitted to use it during WCS administration. The scoring of the WCS was adapted to allow the inclusion of communication acts generated by the child using an augmentative communication device.

The play sessions were coded centrally by a small set of raters. Each sample was scored by a single rater randomly assigned from a pool of 5 raters, all of whom were trained to fidelity (e.g., intraclass correlation coefficient [ICC] ≥ 0.80 across all variables of interest). Approximately 10% of WCS samples (*n* = 80) were then randomly selected and coded by a second rater to determine interrater agreement. These 80 samples came from 11 of the participating FXLEARN sites, with the 2 sites not represented being low-enrolling sites. ICC estimates and their 95% CIs were calculated based on a 2-way random-effects model with absolute agreement averaged across measures. ICC estimates across all reported values were within the “excellent” reliability range for the structured portion of the session (ICC = 0.96, 95% CI = 0.94 to 0.98), unstructured/free play portion of the session, (ICC = 0.98, 95% CI = 0.97 to 0.99), and the WCS total score (ICC = 0.98, 95% CI = 0.96 to 0.99).

Secondary and exploratory measures included the WCS Structured and Unstructured scores, the MSEL DQ and Expressive Language raw score, the Vineland-3 Adaptive Behavior Composite and Communication raw score, the PLS-5 Expressive Communication raw score, the MacArthur-Bates CDI number of words on the Words and Sentences subtest, and the fraction of responders on the CGI-I for Overall Function score. Exploratory outcomes included all other subtest raw scores from the MSEL, Vineland-3, and PLS-5, the 6 subscale scores from the ABC_FX_, the VAS for Language/Communication and for Behavior, and the CGI-I for Language and CGI-S for Language and Overall Function scores. These are all standard measures, which are further described in Supplemental Methods. Exploratory biomarker measures (also described in Supplemental Methods) were outputs from an auditory ERP paradigm, computerized eye-tracking and pupillometry and blood markers of *FMR1* genotype, *FMR1* mRNA, FMRP, and FMRP-regulated proteins.

PK assessment of AFQ056 was done at the 2-, 8-, 10-, and 16-month visits. Plasma was sent to Veeda Clinical Research Ltd. (Ahmedabad, India), where AFQ056 concentration was determined by ultraperformance liquid chromatography–electrospray tandem mass spectrometry by comparison with known standards.

### COVID-19 pandemic adaptations.

Some protocol modifications were made due to the COVID-19 pandemic, including allowance of remote administration of the WCS, adjustment of some toys for the WCS to account for universal masking, and a substitute for the ADOS-2, as this test scoring is not valid with participant and examiner masking. Mandatory in-person visits were limited to those requiring in-person assessments for safety or efficacy outcomes including baseline, month 8 at the end of the placebo-controlled period, and month 16 at the end of the OLE. In-person visits at month 2 and month 10 were conducted if possible. Procedures were put in place to conduct all other visits remotely through telemedicine calls with the site investigator, and collection of parent ratings and WCS administration were also conducted remotely.

Adaptation of the WCS for remote administration included mailing the required testing items to families and then coaching caregivers through administration via Bluetooth-enabled earpieces. Caregivers were instructed to minimize their verbal contributions to interactions during the WCS and to use only the phrases prompted by the remote clinician. Coding of the recorded evaluations was performed in the same manner as in-clinic WCS.

### Statistics.

For the primary, key secondary, and safety outcomes, all analyses were performed according to the ITT principle. Sensitivity analyses of the primary objective were also conducted using a per-protocol population, which included participants who had no major protocol deviations and at least 1 compliant postbaseline PK sample, confirming participants were receiving drug as expected (detectable levels for AFQ056 participants and no detectable levels for placebo participants). For all randomized participants, baseline demographics and clinical characteristics were summarized by treatment group and assessed for differences using the appropriate statistical tests (*t* test/Wilcoxon’s rank-sum test for continuous measures, and χ^2^/Fisher’s exact test for categorical variables).

Three variables were computed to determine whether there was a difference between the treatment groups on language-intervention success. To capture the level of parent participation, a single score for Parent Participation in Language Intervention was computed that summed together the total number of completed coaching, homework, and feedback sessions. The Frequency of Language Intervention Strategy Use, an indirect measure of parent engagement outside of training, was assessed at each session and rated on a scale of 1 to 5 (1 = not at all, 2 = 1 to 2 times, 3 = 3 to 5 times, 4 = 6 to 8 times, 5 = more than 8 times). For each participant, the score was averaged over all complete sessions from the start of language intervention to the end of the placebo-controlled period (54 sessions). The Language Intervention Strategy Rating, a measurement of clinician ratings of how well the parents had learned the strategy, represented a mean rating of both coaching and homework scores from the start of language intervention to the end of the placebo-controlled period. Each item (quality of strategies learned, enthusiasm, confidence, and comfort level for both coaching and homework sessions) on the form was scored on a 1 (lowest) to 7 (highest) scale. Within each session, a mean score of all completed items was computed, and a mean of all completed sessions gave a single score on a 1 to 7 scale.

The primary objective in FXLEARN was to determine whether or not greater improvement in language occurred in young children with FXS treated with AFQ056 in combination with PILI relative to those treated with the PILI and placebo. A longitudinal model was used to estimate the differences in the change of WCS over time for each group. Based on plots of the residuals, heteroscedasticity was present, as illustrated by a fan shape and caused by the skewed distribution of WCS scores. A log base 10 transformation was implemented, and the log of the total WCS was modeled as the outcome. To reduce potential missing scores at baseline, where only 1 of the 2 component scores (structured or unstructured) was missing, the observed component score was directly used to impute the missing component score. However, if both scores were missing, the baseline total score was considered missing. The model included covariates for randomization strata (3 to 4 years or 5 to 6 years), time in months, treatment group, and an interaction between months and treatment group, with the assumption that data were missing at random. Akaike’s Information Criterion (AIC) was used to determine the inclusion of random slopes in addition to random intercepts. The primary comparison using the final model was the estimated difference in change over 8 months between AFQ056 and placebo.

To bolster confidence in the results of the primary analysis, several methods to address the impact of missing data were performed, including an analysis using only observed baseline data, LOCF, and 2 different multiple imputation methods (multiple imputation with treatment-based imputation and a pattern-mixture model with placebo-based imputation).

The key secondary objective was to show greater improvement in specific standardized language, cognitive, and adaptive measures in the combination AFQ056/language intervention group relative to the placebo/language intervention group. The secondary outcomes MSEL, Vineland-3, and PLS-5, as well as the exploratory outcome ABC_FX_, produced numeric scores that were assessed in a manner similar to that described for the primary end point. The number of words spoken from the MacArthur-Bates CDI was expected to have an excess amount of 0 values due to the possibility of nonverbal FXS participants. Therefore, it was prespecified that a zero-inflated Poisson mixed model (ZIPMM) would be used to account for this as well as the repeated measures (54, 55). However, due to the relatively small number of 0 counts observed in the data, the ZIPMM led to unstable estimates for the 0 part of the model, indicating that a less complex negative binomial mixed model may be more appropriate. Given that estimates for the comparison of the average number of words produced was similar between the 2 models, results were reported from the negative binomial mixed model, which was adjusted for time and age strata at randomization. For the CGI-I Overall Function secondary outcome score, participants with a rating of very much improved or much improved were classified as responders and the percentage of positive responders was compared between treatment groups across time. A generalized estimating equations (GEEs) longitudinal logistic regression model was used to model the log odds of a positive response while adjusting for age strata at randomization.

An additional subgroup analysis was done for the WICS, MSEL, and ABC_FX_ using the base model as described above, but including a 3-way interaction among time, treatment, and baseline-functioning status (high functioning was defined as ≥50 on the baseline WCS; low functioning was defined as <50 on the baseline WCS). All primary, key secondary, and exploratory results are reported as point estimates and 95% CIs without adjustment for multiple comparisons.

To determine long-term safety of AFQ056 in this cohort of young children with FXS, the percentages of participants in each group with an AE and the overall rate of AEs were compared using a logistic regression model and a Poisson regression model, respectively, each adjusting for age strata at randomization. These models were repeated to compare AEs within each MedDRA system organ class (SOC) between treatment groups. Any significant differences found within an SOC were further tested by comparing groups across the included MedDRA preferred terms.

Data analysis was primarily performed using SAS statistical software, version 9.4 (SAS Institute Inc.). The ZIPMM models were analyzed using R statistical software (56) functions in the package GLMMadaptive (57).

### Study approval.

One or more legal guardians signed informed consent for study participation of each subject. The study was approved by the central IRB at Massachusetts General Hospital working with NeuroNEXT. Records of central IRB approval were submitted to local IRBs at participating sites.

### Data availability.

Data and associated materials used in the preparation of this article reside in the NIH-supported NIMH Data Repository (NDA, https://nda.nih.gov/study.html?id=2217), which can be accessed with an NDA data access request. Data from participants who did not consent to share data with NDA for future research are not available. Values for all data points in graphs are reported in the Supporting Data Values file. A Manual of Procedures, including training for clinicians, was created for this study and is available upon request.

## Author contributions

EBK, LA, RH, CAE, DH, WEK, and KS conceived the project. EBK, LA, RH, CSC, CAE, LE, FT, DH, WEK, KF, LB, AH, KS, JL, JVV, and AM contributed to the design of methodology. EBK, LA, RH, CAE, DH, LE, FT, WEK, KF, LB, AH, JVV, NeuroNEXT FXLEARN investigators, coordination center investigators, and staff performed experiments. EBK, LA, RH, CSC, and M Cudkowicz acquired funding. EBK, LA, RH, CSC, M Cudkowicz, CAE, JVV, KF, LB, AH, DK, MM, BH, DE, and BP performed project administration. EBK, LA, RH, CSC, CAE, DH, WEK, LB, KS, DK, JL, JF, EK, DE, M Costigan, and TH performed statistical analysis. EBK, LA, RH, DH, M Cudkowicz, LB, AH, DE, and BP supervised the project. EBK, LA, RH, M M Cudkowicz, CAE, DH, LE, FT, WEK, KF, LB, AH, DK, MM, BH, M Costigan, TH, BP, and JVV contributed to drafting the manuscript and provided critical review.

## Supplementary Material

Supplemental data

Trial reporting checklists

ICMJE disclosure forms

Supporting data values

## Figures and Tables

**Table 5:**
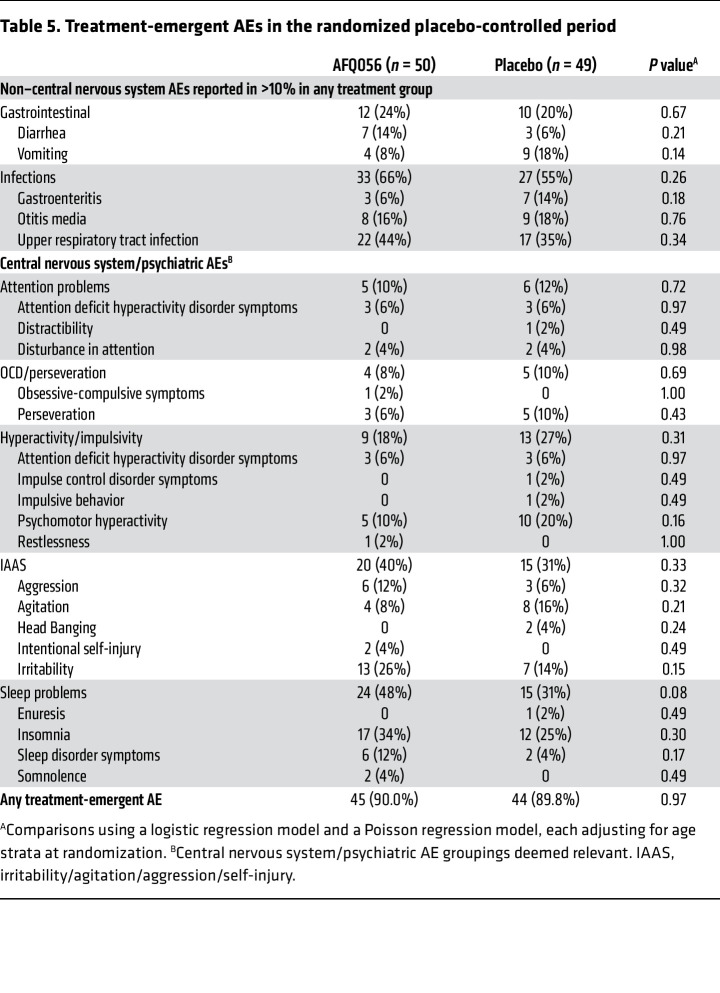
Treatment-emergent AEs in the randomized placebo-controlled period

**Table 4:**
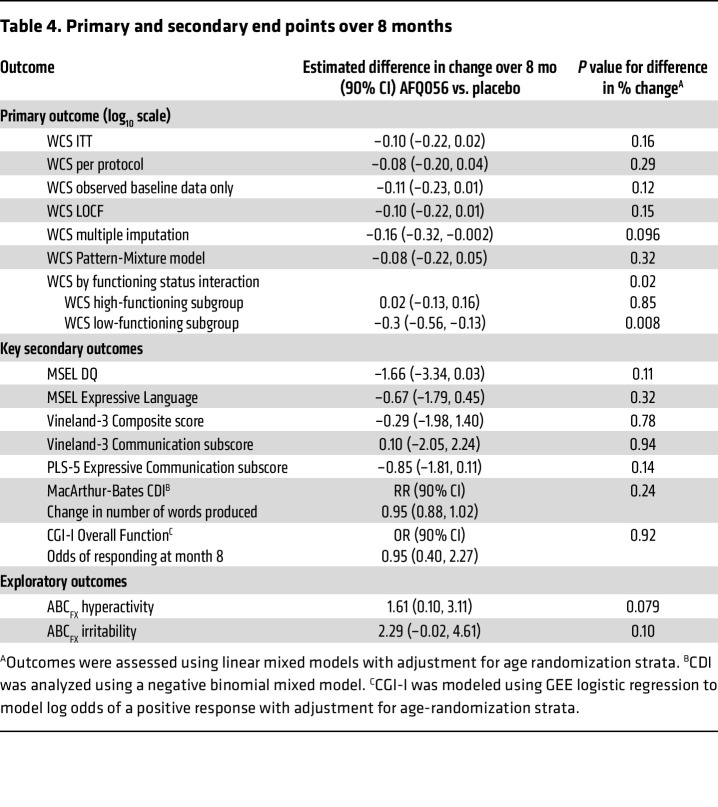
Primary and secondary end points over 8 months

**Table 3:**
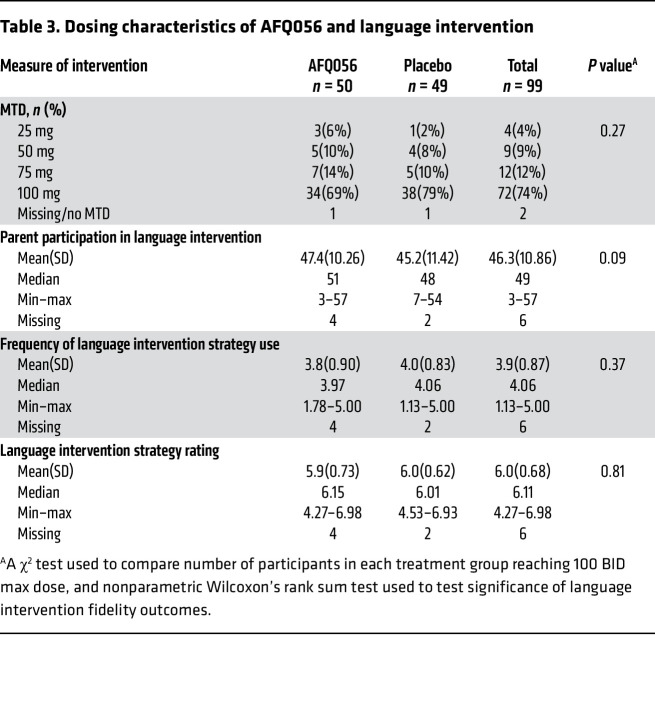
Dosing characteristics of AFQ056 and language intervention

**Table 2:**
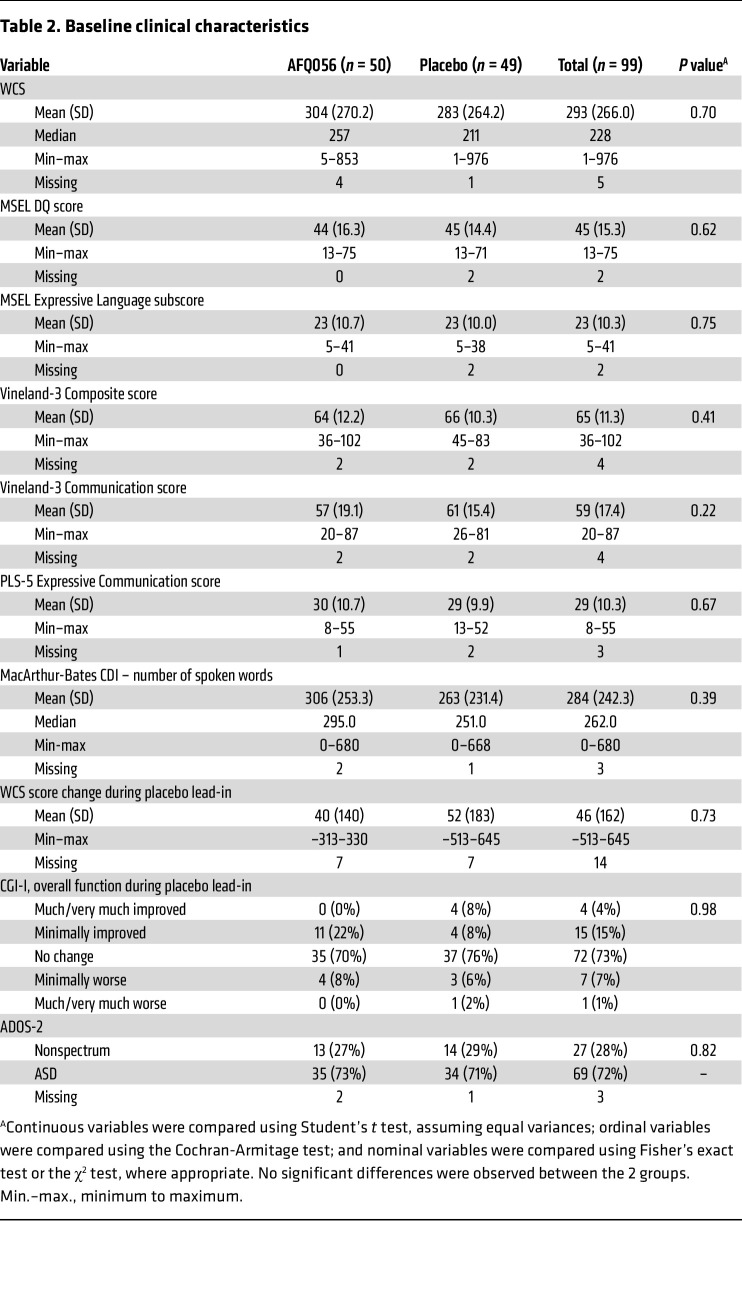
Baseline clinical characteristics

**Table 1:**
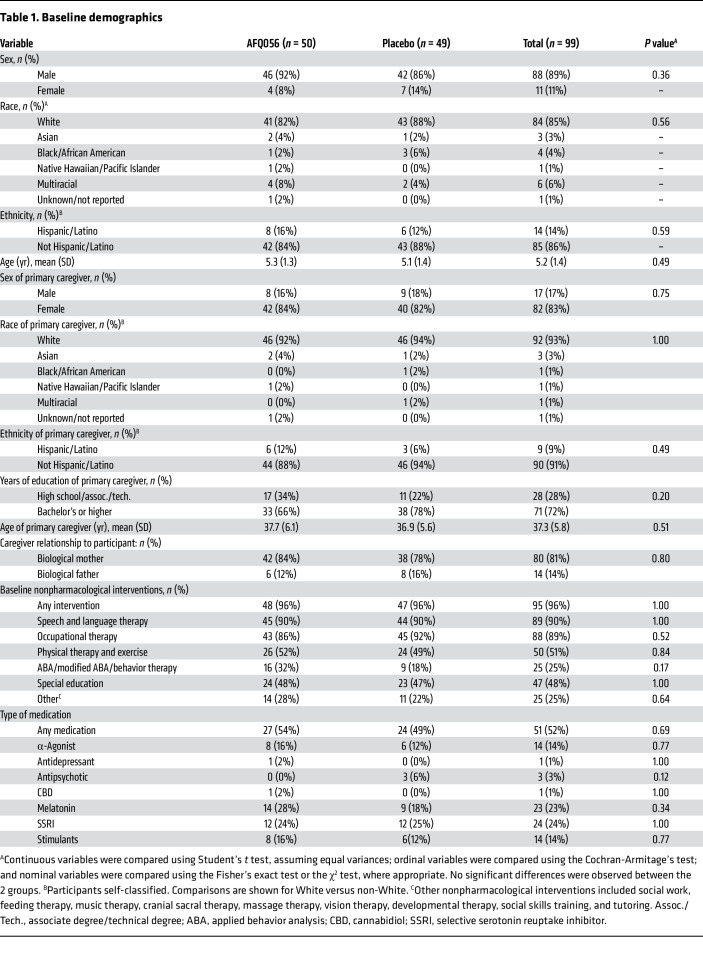
Baseline demographics

## Version 1 08/31/2023

In-Press Preview

## Version 2 03/01/2024

Electronic publication
