# Supplementary material for: Effects of AFQ056 on language learning in fragile X syndrome
Source: J Clin Invest. 2023 Aug 31;134(5):e171723. doi: 10.1172/JCI171723 (PMC10904045; doi:10.1172/JCI171723)
Supplement: Supplemental data [file jci-134-171723-s049.pdf]

Supplemental Acknowledgements:

NeuroNEXT FXLEARN Investigator List

Elizabeth Berry-Kravis, Rush University Medical Center, Chicago, IL

Craig A. Erickson, Cincinnati Hospital and Medical Center, Cincinnati, OH

Robin Filipink, Children’s Hospital of Pittsburgh, Pittsburgh, PA

Andrea Gropman, Children’s National Medical Center, Washington DC

Randi Hagerman, University of California Davis, MIND Institute, Sacramento, CA

Lenora Lewald, Nationwide Children’s Hospital, Columbus, OH

Angela Maxwell-Horn, Vanderbilt University Medical Center, Nashville, TN

Stephanie Morris, Washington University Medical Center, Saint Louis Children’s Hospital, St. Louis, MO

Amanda Palladino Bennett, Children’s Hospital of Philadelphia, Philadelphia, PA

Lisa Prock, Boston Children’s Hospital, Boston, MA

Amy Talboy, Emory University Medical Center, Atlanta, GA

Nicole Tartaglia, Children’s Hospital of Colorado, Denver, CO

Jeremy Veenstra-VanderWeele, New York Presbyterian Hospital, Columbia University, New York, NY

NeuroNEXT Study Site PIs and Grant numbers

U24NS107200 (Tracy Glauser, University of Cincinnati), U24NS107209 (Craig McDonald, University of California Davis), U01NS077323 (Timothy Vollmer, University of Denver), U24NS107183 (Basil Darras, Boston Children's Hospital), U24NS107168 (Karen Marder, Columbia University), U24NS107128 (Beth Malow, Vanderbilt University Medical Center), U24NS107199 (Ramon Diaz-Arrastia, University of Pennsylvania), U24NS107198 (David Clifford, Washington University St. Louis School of Medicine), U24NS107166 (Paula Clemens, University of Pittsburgh Medical Center), U10NS077368 (Roger Packer, Children's National Medical Center), U01NS077366 (Steward Factor, Emory University), U24NS107205 (Stephen Kolb, Nationwide Children's Hospital)

NeuroNEXT Study Site Coordinators, Speech Language Pathologists and Central WCS Raters

Coordinators: Anna DeSonia, Claire Michalak, Kathryn Kudlacz, Hilary Rosselot, Peggy Clark, Angela Molloy, Nicole McCoy, Grace Leonardi, Daniel Woo, Mya Jones, Caitlin Mayer, David Derkacs, Laura Potter, Sumra Afzal, Erica Goude, Bella McLennan, Catie Clyde, Lindsay Ruiz-Graham, Courtney Clark, Megan Jones, Ericka Bickel, Habib Fini, Randev Sandhu, Michelle Cregan, Nanastasia Welnick, Gina VanderVeen, Ester Hamo, Melissa Schneider, Varun Seth, Sophia Deklotz, Hannah Rossing, Maria Abila, Katherine Pawlowski, Audrey Ellis, Stephanie Brewster, Uzma Shamshad, Michelle Coughlin, Evi Abada, Sofia Georghiou, Elizabeth Aaron, Joyce Ann Moran, Lauren Goodwin Shuffrey, Micah Davoren, Grace Lee, Lamia Abbas, Miranda Zuniga-Kennedy, Rebecca Kaiser, Karen W Adkins, Kelly Lowen, Sarah Marler, Emma Dimartini, Sally Furukawa, Tricia McKinney, Amanda Wyatt, Nate Smyk, Elizabeth Harders, Priyanka Kalyani, Erin Reckner, Sunitha Batchu-Vara, Samantha Ferrante, Olga Novak, Mengesha Teshome, Katleen Black, Esinam Wash, Kristine Hall, Christine Amity, Tracy Bui, Grace Tom, Anthony Arnette, Kinaya Hardie, Bergen Kassoff, Jean Luan McColl, Allison Foley, Ami Rosen, Keirsa Nimmons, Rachel Heffern, Christine Baker, Shelli Farley, Ashley Young, Alexandria Alfarano, Brittny Manos, Jessica Fritter, Amy Bartlett, Sarah Heintzman, Karen Carter, Andrea Debs, Sarah Borror, Christina Waibel.

Speech Language Pathologists: Kristen Vincent, Katherine Eichstadt, Liz Kirkpatrick, Sandra Grether, Bridgitt Pauly, Dawn Girten, Jennifer Vannest, Amy Banasik, Amy Shafer, Rachel Reetzke, Teresa Hall, Hope Dickinson, Kara Corley, Christine Larned, Carly Feiner, Charles Hausman, Lea Helen Evans, Julie Weaver, Lisa Reale, Laura Ball, Jasmine Stevens, Stephanie Nixon, Jamie Boster, Kelliann Calabria, Samantha Steiner.

Central WCS Raters: Sarah Nelson, Robyn Tempero Feigles, Emily McFadd, Amanda Dimachkie Nunnally.

Supplemental Methods

Includes:

Supplementary Standardized Clinical Measure and Biomarker Descriptions

Supplementary Tables

Supplementary Table 1: Table S1. Phenotypes Reversed by Correction of Excessive mGluR5 Signaling in Fragile X Models

Supplementary Table 2: Table S2. Treatment-Emergent Adverse Events During Open Label Extension

Supplementary Table 3: Table S3. Schedule of Activities for FXLEARN Trial

References Cited Only in Supplementary Materials (58-115, references cited in supplementary materials as well as the main paper are in the list of references in the manuscript)

Supplementary Standardized Clinical Measure and Biomarker Descriptions

Standardized Clinical Measures

Mullen Scales of Early Learning (MSEL) (58) includes five subscales: gross motor, fine motor, visual reception, expressive language, and receptive language, and an early learning composite (ELC). The MSEL was standardized for children ages 3 months to 68 months; in addition to standard scores, it also provides age equivalents. In this study, raw scores and age equivalent scores were used. Additionally, instead of using the ELC standard score, which is not available for children over age 68 months and not always accurate in populations with ID, DQ was calculated from age equivalent scores. Participants were required to obtain a DQ lower than 75% of their chronological age at Screening to be eligible for the study. The MSEL has been utilized in studies with young children with FXS for many years to track developmental progress (59-61). The MSEL was administered at visits 1, 3, 5, 9, and 14 (Screening, Baseline, months 2, 8, and 16) by the same examiner for a given participant as possible throughout the trial.

Preschool Language Scales–5th Edition (PLS-5) (62) is a comprehensive standardized developmental language assessment that is normed for use with children from birth through age 7 years (up to 8th birthday). This test requires pointing to or verbally responding to items and yields raw scores, percentile ranks and standard scores for auditory comprehension and expressive communication as

well as a total language scores. This measure has been used to track language progress of children with FXS in longitudinal studies and to describe the language profile of patients with FXS for many years (63, 64). Raw scores were used for this study. The PLS-5 was administered at visits 1, 3, 5, 9, 11 and 14 (Screening, Baseline, months 2, 8, 10 and 16) by the same examiner for a given participant as possible throughout the trial.

MacArthur-Bates Communicative Development Inventory (CDI) (65) (Words and Sentences version) consists of two parts: a) Part 1 (Words Children Use) is a 680-word vocabulary checklist in which the parent indicates those vocabulary words the child regularly produces in spoken language and b) Part 2 (Sentences and Grammar) assesses the child's expression, several aspects of grammar and word endings. This parent-report measure has been well-validated against direct observation and assessment measures of child language status and growth and has been shown effective in characterizing language change in populations with limited communication abilities (66-69). Parents used the same test booklet at each time point, updating their previous responses using different color ink to indicate newly acquired words or grammatical constructions. Raw scores for Words and Sentences/Grammar Subscales were used. The MacArthur-Bates CDI was administered at visits 1, 3, 5, 7, 9, 11, 12, 14 and 15 (Screening, Baseline, months 2, 4, 8, 10, 12, 16 and Follow Up).

Vineland Adaptive Behavior Scales, Third Edition (Vineland-3) (70) was administered as an interview to a parent/caregiver by trained study staff authorized to perform the interview. The Vineland-3 is a valid and reliable standardized measure of a person's adaptive level of functioning from birth to 90 years of age. The Vineland-3 yields a composite score and domain standard scores in the domains of Communication (receptive, expressive, and written adaptive language functions), Daily Living Skills (personal, domestic, and community skills), Socialization (interpersonal relationships, play and leisure time, and coping abilities), and Motor Skills (gross and fine motor skills). The Vineland-3 have been used extensively to track progress of children with FXS in large longitudinal studies and clinical trials (71). For this study, the composite standard score and all the 4 domain raw scores were used. The Vineland-3 was administered at visits 1, 3, 5, 9, 11 and 14 (Screening, Baseline, months 2, 8, 10, 16 and Follow Up) by the same interviewer for a given participant as possible throughout the trial.

Visual Analog Scales (VAS) for Language and Behavior was completed at Screening and at all subsequent study visits by the parent/caregiver, who marked their child's overall language ability and behavioral function on separate visual lines measuring 10 cm with one side marked "worst language" (or "worst behavior") and the other side marked "best language for age" (or "best behavior"). This allowed parent-perceived improvements or worsening of language and behavior over the treatment period to be evaluated. Scores were centimeter distance from the "best language" or "best behavior" end of the line.

Aberrant Behavior Checklist-Community Edition (ABC-C) is a parent/caregiver report measure is the gold standard measure of problem and interfering behaviors in clinical trials in DD (72) and is a FDA-vetted endpoint utilized in the approvals of risperidone

and aripiprazole for irritability in youth with ASD and in virtually all prior FXS clinical trials (41). The ABC-C was subjected to validation and factor analysis based on ratings of over 600 individuals with FXS and found to factor into 6 domains instead of 5 (24). Scores were analyzed using the FXS-specific algorithm (ABC_{FX}) such that 55 of the items resolve into 6 subscales (Irritability, Lethargy, Social Avoidance, Stereotypic Behavior, Hyperactivity, and Inappropriate Speech) (24). Scores for each of the six subscales were used in this study. The ABC_{FX} was administered at all study visits.

Clinical Global Impression–Improvement (CGI-I) Scales (73) for Language and Overall Function were rated by the investigator at all study visits after Screening. CGI-I ratings were performed relative to Screening through the Baseline visit. After the Baseline visit, CGI-I ratings were performed relative to Baseline. Completion of this scale requires the clinician to rate how much the participant's illness has improved or worsened relative to a baseline state. The CGI-I is a seven-point scale that includes the following ratings: 1 = very much improved, 2 = much improved, 3 = minimally improved, 4 = no change, 5 = minimally worse, 6 = much worse, 7 = very much worse. The CGI-I (and CGI-S, see below) are gold standard global measures of severity and change with treatment in placebo-controlled pharmacotherapy trials in FXS and developmental disabilities. Ratings were given separately for Language and Overall Function. An anchoring document/rating guide was provided to ensure consistent ratings; investigators also took part in a fidelity training.

Clinical Global Impression–Severity (CGI-S (73) Scales for Language and Overall Function were rated by the investigator at all study visits. The CGI-S is a 7-point scale that requires the clinician to rate the severity of the participant's illness at the time of assessment, relative to the clinician's past experience with participants who have the same diagnosis. Considering the rater's total clinical experience, a participant is assessed on severity of illness using the following classifications: 1 = normal, not at all ill; 2 = borderline ill; 3 = mildly ill; 4 = moderately ill; 5 = markedly ill; 6 = severely ill; or 7 = extremely ill. Ratings were performed separately for Language and Overall Function. An anchoring document/rating guide was provided to ensure consistent ratings as in prior studies using the measure in FXS (74); investigators also took part in a fidelity training.

Biomarkers

Auditory ERP using an oddball paradigm with stimuli delivered based on a protocol previously reported in children with FXS (75), preceded by 3 minutes of resting state EEG, as a direct index of brain function, was performed at 8 sites brought to fidelity for data collection, at Screening, Baseline and 2, 8, 10, 16 month and Follow Up visits (visits 1, 3, 5, 9, 14, and 15). Raw electrophysiological data was analyzed centrally (University of Oklahoma).

Computerized Eye-Tracking and Pupillometry was done with the subjects at Screening, AFQ Baseline and 2, 8, 16 month and Follow Up visits (visits 1, 3, 5, 9, 14, and 15). A computerized eye-tracking device (Tobii) was used applying a standardized protocol

developed found to have good reproducibility in FXS (76, 77). Automated assessments of time spent looking at and number of fixations on regions of interest (e.g., eyes, nose, mouth) in face images was collected, as well as changes in pupil diameter associated with viewing of faces, the latter as an index of autonomic arousal.

Blood Biomarkers were collected at Screening, 2, 8, and 16 month visits (visits 1, 5, 9, and 14). Blood biomarkers analyzed included *FMR1* genotyping (only collected at one visit), *FMR1* mRNA, FMRP, and other markers known to be key proteins in the signaling pathways regulated by FMRP and to be abnormal in FXS including ERK (78), Akt (79), S6 kinase (80), APP (81), and MMP9 (82).

Supplementary Tables

Supplementary Table S1. Phenotypes Reversed by Correction of Excessive mGluR5 Signaling in Fragile X Models*

Phenotypes	Examples	References
Molecular/cellular phenotypes	signaling pathway activity, dendritic protein synthesis, α-amino-3-hydroxy-5-methyl-4-isoxazolepropionic acid (AMPA) receptor internalization, dendritic spine morphology	(15), (83), (84), (85), (86), (87), (88), (89)**, (90), (91), (92)**, (93), (94)
Plasticity/synaptic/electrophysiologic	LTP/LTD, audiogenic seizures, network hyperexcitability – UP states, prepulse inhibition	(13), (17)**, (19)**, (33)**, (88), (93)**, (95), (96), (97), (98)**, (99), (100), (101), (102)**, (103), (104)**, (105)**, (106)**, (107)**
Behavioral	open field hyperactivity, social novelty	(18), (85), (93), (106)** , (108)**, (109), (110), (111)**, (112)**, (113)**
Cognitive	object recognition, inhibitory avoidance, maze learning	(18), (19)**, (86), (87), (94), (101), (108)**, (109), (114)
Physical	growth trajectory, macro-orchidism	(108)**, (115)

*This Table provides representative examples indicating the extensive body of research on correction of mGluR5 signaling by mGluR5 NAMs or genetic/molecular corrections in FXS models, but is not meant to be a comprehensive list
**In these studies investigators were blinded to treatment groups

Supplementary Table S2. Treatment-Emergent Adverse Events During Open Label Extension

	**AFQ056 (N=43)**	**Placebo (N=46)**	**p-value**
**Non-Central Nervous System AEs Reported in >10% in Any Treatment Group, reported as N (%)**			
**Gastrointestinal**	**13 (30%)**	**11 (24%)**	**0.48**
Diarrhea	5 (12%)	5 (11%)	0.88
Vomiting	6 (14%)	5 (11%)	0.66
**Infections**	**28 (65%)**	**24 (52%)**	**0.21**
Otitis Media	3 (7%)	5 (11%)	0.52
Upper Respiratory Tract Infection	22 (51%)	19 (41%)	0.36
**Central Nervous System/Psychiatric AEs (All), N (%)**	**16 (37%)**	**27 (59%)**	**0.04**
**Attention Problems***	**3 (7%)**	**4 (9%)**	**0.73**
**OCD/Perseveration***	**0**	**1 (2%)**	**1.00**
Stubbornness	0	1 (2%)	1.00

	AFQ056 (N=43)	Placebo (N=46)	p-value
**Hyperactivity/Impulsivity***	**11 (26%)**	**17 (37%)**	**0.21**.73
Attention Deficit Hyperactivity Disorder Symptoms	3 (7%)	4 (9%)	0.48
Impulsive Behavior	1 (2%)	0	0.05
Psychomotor Hyperactivity	8 (19%)	17 (37%)	
**IAAS***	**9 (21%)**	**15 (33%)**	**0.23**
Aggression	3 (7%)	4 (9%)	0.79
Agitation	4 (9%)	1 (2%)	0.16
Head Banging	0	0	N/A
Intentional Self-Injury	0	0	N/A
Irritability	3 (7%)	10 (22%)	0.06

	**AFQ056**<br><br>**(N=43)**	**Placebo**<br><br>**(N=46)**	**p-value**
**Sleep Problems***	**5 (12%)**	**15 (33%)**	**0.02**
Enuresis	0	1 (2%)	1.00
Insomnia	4 (9%)	14 (30%)	0.02
Lethargy	1 (2%)	0	0.48
Sleep Disorder Symptoms	0	1 (2%)	1.00
Somnolence	0	1 (2%)	1.00
**Any Treatment-Emergent AE**	**36 (83.7%)**	**40 (87.0%)**	**0.70**

*Central nervous system/psychiatric AE groupings deemed relevant by investigators.

Supplementary Table S3. Schedule of Activities for the FXLEARN Trial

Evaluation	Screening (Month -4, v1)	Month[3] -2 (v2)	Random-ization/ Baseline (Month 0, v3)	Months (visit #)[3] 1 (v4)[20]	2 (v5)	3 (v6)[20]	4 (v7)	6 (v8)	8 (v9)	9 (v10)[20]	10 (v11)	12 (v12)	14 (v13)	16[16] (v14)	Follow up (Month 17, v15)[16]
Written Informed Consent	X														
Inclusion/Exclusion Review	X	X	X	X	X	X	X	X	X	X	X	X	X	X	X
Documentation of Disease/Disorder	X														
Medical History/Demographics	X														
Autism Diagnostic Observation Scale		X[11]													
Vital Signs: HR, BP (sitting)	X	X	X	X	X	X	X	X	X	X	X	X	X	X	X
Weight	X	X	X	X	X	X	X	X	X	X	X	X	X	X	X
Height	X	X	X	X	X	X	X	X	X	X	X	X	X	X	X
Physical & Psychiatric Examination	X	X	X	X	X	X	X	X	X	X	X	X	X	X	X
Neurological Examination	X	X	X	X	X	X	X	X	X	X	X	X	X	X	X
Funduscopic Examination[8]	X	X	X		X		X		X		X	X		X	X
EKG	X[15]				X				X		X			X	
Concomitant Medication/Therapy Review	X	X	X	X	X	X	X	X	X	X	X	X	X	X	X
Safety Labs[1]	X[17]				X				X		X			X	
Research Labs for Biomarkers[2]	X[13]				X				X		X			X	
Blood PK Sampling	X[13]				X				X		X			X	
Urine dipstick	X[14]				X				X		X			X	
Randomization[12]			X												
**Evaluation**	**Screening (Month -4, v1)**	**Month[3] -2 (v2)**	**Random-ization/ Baseline (Month 0, v3)**	**Months (visit #)[3] 1 (v4)[20]**	**2 (v5)**	**3 (v6)[20]**	**4 (v7)**	**6 (v8)**	**8 (v9)**	**9 (v10)[20]**	**10 (v11)**	**12 (v12)**	**14 (v13)**	**16[16, 17] (v14)**	**Follow up (Month 17, v15) [17]**
Dispense Study Drug	X	X	X		X		X	X	X		X	X	X	X	

Dose Review/Titration[4]			X	X					X	X				X[5]	
Drug Accountability/Compliance[18]		X	X		X		X	X	X		X	X	X	X	X
Adverse Event Review		X	X	X	X	X	X	X	X	X	X	X	X	X	X
Suicidality Screen	X	X	X	X	X	X	X	X	X	X	X	X	X	X	X
Implement/Review Language Intervention/Compliance[6]				X[10]	X	X	X	X	X	X	X	X	X	X	X
Weighted child intentional communication score (WCS)	X		X		X		X	X	X		X	X		X	X
Auditory ERP[7]	X[15]		X		X		X		X					X	X
Mullen Scales of Early Learning (MSEL)	X		X		X				X					X	
Vineland Adaptive Behavior Scale-3 (Vineland-3)	X		X		X		X	X	X			X		X	X
Preschool Language Scale – 5 (PLS-5)	X		X		X				X					X	
MacArthur Vocabulary Scale	X		X		X		X	X	X			X		X	X
VAS-Language	X	X	X	X	X	X	X	X	X	X	X	X	X	X	X
CGI-I[9]		X	X	X	X	X	X	X	X	X	X	X	X	X	X
CGI-S[9]	X	X	X	X	X	X	X	X	X	X	X	X	X	X	X
VAS-Behavior	X	X	X	X	X	X	X	X	X	X	X	X	X	X	X
ABC_{FX}	X		X		X		X	X	X		X	X		X	X
Eye Tracking[7]	X[15]		X		X		X		X					X	X
Children's Sleep Habits Questionnaire	X		X	X	X				X	X	X			X	X

1. CBC, Comprehensive Metabolic Profile (CMP), TSH at screening, 2, 8, 10, 16 months.
2. Cellular biomarkers: ERK, AKT, S6 kinase, APP, and MMP9 at screening, 2, 8, 10, 16 months; DNA banking at screening; RNA; protein; serum banking at screening, 2, 8, 10, 16 months.
3. Visit windows were ±5 days for v2-v3 based on the date of Visit 1, and ±5 days for v4-v15 based on date of visit 3.
4. Dose titration done weekly from the baseline visit (v3) to the 2 months visit (v5) and from the 8 months visit (v9) to the 10 months visit (v1).
5. Wean over 1-2 weeks for dose >25 mg.
6. The 2 months visit corresponded to the language intervention baseline assessment visit and, in addition to monitoring of the language intervention at study visits, video-conferencing calls was done with the language therapist to monitor and for ongoing training with the language intervention weekly for 4 months with a ±2 day window between v5 –v8, then monthly with a ±7 day window for the duration of the trial.
7. As possible (expected >50% of cohort).
8. The PI (a FXS specialist) performed the funduscopic examination. Many of the study assessments depended on the child's visual function and constituted functional vision assessments. All clinicians and assessors will be trained to observe for evidence of visual compromise during other routine

visits and assessments. If any evidence of visual compromise was noted during any of these assessments, the patient was sent for an ophthalmology exam for further evaluation.

9. For this study two sets of CGI-S/CGI-I were used. The Language/Communication CGI-S/CGI-I was anchored to language development. The Overall Function CGI-I/CGI-S took into account all areas of function including cognition, adaptive behavior, and maladaptive behavior and was anchored to the child's overall functioning in the home and environments outside the home relative to a typical child of the same age.
10. SLPs set up 1-2 distance technology training sessions with the subject family during the first month of the trial (after visit 4 – Month 1). During the call, SLPs reviewed the technology procedures, worked with the parent/primary caregiver to set up the intervention environment and worked on setting up the equipment as necessary for the language intervention. These practice calls needed to be completed prior to visit 5 (Month 2), after which the language intervention began.
11. The ADOS could be administered at any point between Screening and Baseline (i.e., visits 1, 2 or 3), but was done prior to randomization.
12. Subjects should have been at least 3 years old at the time of randomization.
13. If the PK and biomarker blood samples could not be collected at the screening visit, sites collected them at the -2 months or baseline visits (v2 or v3) as long as it was prior to randomization. If the PK and biomarker samples could not be obtained at a visit following randomization (v5, 9, 11 and 14), then the sample could be re-drawn at the next visit.
14. Urine dipstick testing was completed during the screening visit when possible. If the urine dipstick testing could not be completed at the screening visit, sites collected them at the -2 months or baseline visits (v2 or v3) as long as it was prior to randomization. If the urine dipstick testing could not be obtained at a visit following randomization (v5, 9, 11 and 14), then the sample was collected at the next visit.
15. If the auditory ERP, eye tracking, and/or EKG could not be administered at the screening visit, sites administered them at the -2 months visit (v2).
16. For subjects with an open label extension that was less than 6 months, month 16 (Visit 14) occurred following completion of the open label extension. The length of the open label extension, and the number of months between Visit 11 and Visit 14, was determined based on when the subject was consented and their dose in the open label extension. For those for whom the open label extension was shortened, all study visits occurred at the usual times in the protocol but visit 14 was done just before February 28, 2021.
17. If a subject was withdrawn from the study during the placebo-lead in period or prior to randomization, they were brought back in for an abbreviated follow-up/termination visit. Subject Withdrawal Criteria for more details. Subjects withdrawing early (after randomization had occurred) completed an end of study visit at which assessments and procedures for the 16 month visit (v14) were conducted, and then if possible completed a follow up visit at which the same procedures as listed for the 17 month follow up visit (v15) were conducted. If subjects discontinue study drug prior to the early termination visit, only safety outcomes were assessed during the termination visit (Blood draw, urine dipstick, EKG, vitals, physical, neurological and psychological exams, suicidality assessment, fundoscopic exam, AE review, study drug accountability) and a follow-up email or phone call to document adverse events were completed 30 days later.
18. If the safety sample obtained at screening visit could not be analyzed, the sample could be re-drawn at visit 2. In this case dosing could begin, but the results from the repeat blood test should have been obtained and were acceptable before randomization could be done at Visit 3. If a lab result was abnormal and deemed not clinically significant by the Site Investigator, dosing could proceed. If the safety samples could not be obtained at a visit following randomization (v5, 9, 11 and 14), then the sample could be re-drawn at the next visit.
19. Depending upon the timing of the visit, the evening/p.m. dose was administered after the completion of the visit. If the visit was done earlier in the day, the family retained the current bottle being used in order to administer the evening dose on the day of visit 3. The bottle diluted at visit 3 will then was used starting with the morning dose the day after the visit. This bottle was then returned at visit 4 so that site study staff could complete accountability documentation.
20. Visit 4 (Month 1), Visit 6 (Month 3) and Visit 10 (Month 9) could be completed remotely if travel to these visits was difficult for the family. All parent rated measures and questionnaires were completed over the phone. Vitals were optional for these visits.

References Cited Only in Supplementary Materials (References 58-115)

58. Mullen EM. *Mullen Scales of Early Learning.* AGS Circle Pines, MN; 1995.
59. Garcia-Nonell C, et al. Secondary medical diagnosis in fragile X syndrome with and without autism spectrum disorder. *Am J Med Genet A.* 2008;146A(15):1911-6. doi: 10.002/ajmg.a.32290.
60. Philofsky A, et al. Linguistic and Cognitive Functioning and Autism Symptoms in Young Children WithFragile X syndrome. *Am J Ment Retard.* 2004;109(3):208-18.
61. Wheeler AC, et al. Emergence of developmental delay in infants and toddlers with an FMR1 mutation. *Pediatrics.* 2021;147(5).
62. Zimmerman I, Steiner V, and Pond R. *Preschool Language Scale-5th Edition.* San Antonio, TX: Pearson; 2011.
63. Hernandez RN, et al. Autism spectrum disorder in fragile X syndrome: A longitudinal evaluation. *Am J Med Genet A.* 2009;149A(6):1125.
64. Kaufmann WE, et al. Autism spectrum disorder in fragile X syndrome: Communication, social interaction, and specific behaviors. *Am J Med Genet A.* 2004;129A(3):225.
65. Fenson L. *MacArthur-Bates Communicative Development Inventories: user's guide and technical manual.* Paul H. Brookes Publishing Company; 2007.
66. Charman T, Drew A, Baird C, and Baird G. Measuring early language development in preschool children with autism spectrum disorder using the MacArthur Communicative Development Inventory (Infant Form). *J Child Lang.* 2003;30(1):213-36.
67. Luyster R, et al. Predicting outcomes of children referred for autism using the MacArthur–Bates Communicative Development Inventory. *J Speech Lang Hear Res.* 2007;50(3):667-87.
68. Mervis CB, and Robinson BF. Expressive vocabulary ability of toddlers with Williams syndrome or Down syndrome: A comparison. *Dev Neuropsychol.* 2000;17(1):111-26.
69. Miller JF, Sedey AL, and Miolo G. Validity of parent report measures of vocabulary development for children with Down syndrome. *J Speech Lang Hear Res.* 1995;38(5):1037-44.
70. Sparrow SS, Cicchetti DV, and Saulnier CA. *Vineland Adaptive Behavior Scales, Third Edition (Vineland-3).* San Antonio: TX: Pearson; 2016.
71. Hoffmann A, et al, et al. Language across the lifespan in fragile X syndrome: Characteristics and considerations for assessment. *Brain Sci.* 2020;10(4):212.
72. Aman MG, Burrow WH, and Wolford PL. The Aberrant Behavior Checklist-Community: factor validity and effect of subject variables for adults in group homes. *Am J Ment Retard.* 1995.
73. Guy W. Clinical Global Impression Scale. The ECDEU Assessment Manual for Psychopharmacology-Revised. 1976;DHEW Publ No ADM 76:218-22.

74. Berry-Kravis E, et al. A double-blind, randomized, placebo-controlled clinical study of trofinetide in the treatment of fragile X syndrome. *Pediatr Neurol.* 2020;110:30-41.
75. Schneider A, et al. Electrocortical changes associated with minocycline treatment in fragile X syndrome. *J Psychopharmacol.* 2013;27(10):956-63.
76. Farzin F, Rivera SM, and Hessl D. Brief report: Visual processing of faces in individuals with fragile X syndrome: An eye tracking study. *J Autism Dev Disord.* 2009;39(6):946-52.
77. Farzin F, et al. Reliability of eye tracking and pupillometry measures in individuals with fragile X syndrome. *J Autism Dev Disord.* 2011;41(11):1515-22.
78. Berry-Kravis E, et al. Open-label treatment trial of lithium to target the underlying defect in fragile X syndrome. *J Dev Behav Pediatr.* 2008;29(4):293-302.
79. Hoeffer CA, et al. Altered mTOR signaling and enhanced CYFIP2 expression levels in subjects with fragile X syndrome. *Genes Brain and Behav.* 2012;11(3):332-41.
80. Gross C, and Bassell GJ. Excess protein synthesis in FXS patient lymphoblastoid cells can be rescued with a p110β-selective inhibitor. *Mol Med.* 2012;18(3):336-45.
81. Westmark CJ. The quest for fragile X biomarkers. *Mol Cell Pediatr.* 2014;1(1):1-4.
82. Dziembowska M, et al. High MMP-9 activity levels in fragile X syndrome are lowered by minocycline. *Am J Med Genet A.* 2013;161(8):1897-903.
83. Aschrafi A, et al. The fragile X mental retardation protein and group I metabotropic glutamate receptors regulate levels of mRNA granules in brain. *Proc Natl Acad Sci USA.* 2005;102(6):2180-5.
84. Busquets-Garcia A, et al. Targeting the endocannabinoid system in the treatment of fragile X syndrome. *Nat Med.* 2013;19(5):603-7.
85. de Vrij FM, et al. Rescue of behavioral phenotype and neuronal protrusion morphology in Fmr1 KO mice. *Neurobiol Dis.* 2008;31(1):127-32.
86. Gandhi RM, Kogan CS, and Messier C. 2-Methyl-6-(phenylethynyl) pyridine (MPEP) reverses maze learning and PSD-95 deficits in Fmr1 knock-out mice. *Front Cell Neurosci.* 2014;8:70.
87. Gross C, et al. Excess phosphoinositide 3-kinase subunit synthesis and activity as a novel therapeutic target in fragile X syndrome. *J Neurosci.* 2010;30(32):10624-38.
88. McBride SMJ, et al. Pharmacological rescue of synaptic plasticity, courtship behavior, and mushroom body defects in a Drosophila model of fragile X syndrome. *Neuron.* 2005;45(5):753-64.
89. Min WW, et al. Elevated glycogen synthase kinase-3 activity in Fragile X mice: key metabolic regulator with evidence for treatment potential. *Neuropharmacology.* 2009;56(2):463-72.
90. Pop AS, et al. Rescue of dendritic spine phenotype in Fmr1 KO mice with the mGluR5 antagonist AFQ056/Mavoglurant. *Psychopharmacology.* 2014;231(6):1227-35.

91. Su T, et al. Early continuous inhibition of group 1 mGlu signaling partially rescues dendritic spine abnormalities in the Fmr1 knockout mouse model for fragile X syndrome. *Psychopharmacology*. 2011;215(2):291-300.
92. Westmark CJ, et al. Reversal of fragile X phenotypes by manipulation of AβPP/Aβ levels in Fmr1KO mice. *PloS One.* 2011;6(10):e26549.
93. Yuskaitis CJ, et al. Lithium ameliorates altered glycogen synthase kinase-3 and behavior in a mouse model of fragile X syndrome. *Biochem Pharmacol.* 2010;79(4):632-46.
94. Bolduc FV, et al. Excess protein synthesis in Drosophila fragile X mutants impairs long-term memory. *Nat Neurosci.* 2008;11(10):1143-5.
95. Auerbach BD, and Bear MF. Loss of the fragile X mental retardation protein decouples metabotropic glutamate receptor dependent priming of long-term potentiation from protein synthesis. *J Neurophysiol.* 2010;104(2):1047-51.
96. Chen T, et al. Pharmacological rescue of cortical synaptic and network potentiation in a mouse model for fragile X syndrome. *Neuropsychopharmacol.* 2014;39(8):1955-67.
97. Choi CH, et al. Pharmacological reversal of synaptic plasticity deficits in the mouse model of fragile X syndrome by group II mGluR antagonist or lithium treatment. *Brain Res.* 2011;1380:106-19.
98. Chuang S-C, et al. Prolonged epileptiform discharges induced by altered group I metabotropic glutamate receptor-mediated synaptic responses in hippocampal slices of a fragile X mouse model. *J Neurosci.* 2005;25(35):8048-55.
99. De Esch C, , et al. Fragile X mice have robust mGluR5-dependent alterations of social behaviour in the Automated Tube Test. *Neurobiol Dis.* 2015;75:31-9.
100. Hays SA, Huber KM, and Gibson JR. Altered neocortical rhythmic activity states in Fmr1 KO mice are due to enhanced mGluR5 signaling and involve changes in excitatory circuitry. *J Neurosci.* 2011;31(40):14223-34.
101. Levenga J, et al. AFQ056, a new mGluR5 antagonist for treatment of fragile X syndrome. *Neurobiol Dis.* 2011;42(3):311-7.
102. Qiu L-F, Limbic epileptogenesis in a mouse model of fragile X syndrome. *Cerebral Cortex.* 2009;19(7):1504-14.
103. Volk LJ, Daly CA, and Huber KM. Differential roles for group 1 mGluR subtypes in induction and expression of chemically induced hippocampal long-term depression. *J Neurophysiol.* 2006;95(4):2427-38.
104. Wang GX, Smith SJ, and Mourrain P. Fmr1 KO and fenobam treatment differentially impact distinct synapse populations of mouse neocortex. *Neuron.* 2014;84(6):1273-86.
105. Xu Z-H, et al. Group I mGluR antagonist rescues the deficit of D1-induced LTP in a mouse model of fragile X syndrome. *Mol Neurodegener*. 2012;7(1):1-14.
106. Yan Q, et al. Suppression of two major fragile X syndrome mouse model phenotypes by the mGluR5 antagonist MPEP. *Neuropharmacology*. 2005;49(7):1053-66.
107. Zhao W, et al. Extracellular glutamate exposure facilitates group I mGluR-mediated epileptogenesis in the hippocampus. *J Neurosci.* 2015;35(1):308-15.
108. Dölen G, et al. Correction of fragile X syndrome in mice. *Neuron.* 2007;56(6):955-62.

109. Franklin AV, et al. Glycogen synthase kinase-3 inhibitors reverse deficits in long-term potentiation and cognition in fragile X mice. *Biol psychiat.* 2014;75(3):198-206.

110. Gantois I, et al. Chronic administration of AFQ056/Mavoglurant restores social behaviour in Fmr1 knockout mice. *Behavioural Brain Res.* 2013;239:72-9.

111. Kramvis I, et al. Hyperactivity, perseveration and increased responding during attentional rule acquisition in the Fragile X mouse model. *Front Behav Neurosci.* 2013;7:172.

112. Pacey LK, Tharmalingam S, and Hampson DR. Subchronic administration and combination metabotropic glutamate and GABAB receptor drug therapy in fragile X syndrome. *J Pharmacol Exp Ther.* 2011;338(3):897-905.

113. Thomas AM, et al. Group I metabotropic glutamate receptor antagonists alter select behaviors in a mouse model for fragile X syndrome. *Psychopharmacology.* 2012;219(1):47-58.

114. Vinueza Veloz M, et al. The effect of an mGluR5 inhibitor on procedural memory and avoidance discrimination impairments in Fmr1 KO mice. *Genes Brain Behav.* 2012;11(3):325-31.

115. Chang S, et al. Identification of small molecules rescuing fragile X syndrome phenotypes in Drosophila. *Nat Chem Biol.* 2008;4(4):256-63.
